# 3D Printing Highly Efficient Ion‐Exchange Materials via a Polyelectrolyte Microphase Separation Strategy

**DOI:** 10.1002/smsc.202400019

**Published:** 2024-03-10

**Authors:** Kenny Lee, Jitendra Mata, Valentin A. Bobrin, Dipan Kundu, Vanessa K. Peterson, Nathaniel Corrigan, Cyrille Boyer

**Affiliations:** ^1^ Cluster for Advanced Macromolecular Design (CAMD) UNSW Australia Sydney NSW 2052 Australia; ^2^ Australian Centre for Neutron Scattering (ACNS) Australian Nuclear Science and Technology Organisation (ANSTO) Lucas Heights NSW 2234 Australia; ^3^ School of Mechanical and Manufacturing Engineering UNSW Sydney Kensington NSW 2052 Australia; ^4^ School of Chemical Engineering UNSW Sydney Kensington NSW 2052 Australia; ^5^ Australian Centre for NanoMedicine (ACN) School of Chemical Engineering UNSW Australia Sydney NSW 2052 Australia

**Keywords:** ion‐exchange materials, nanostructured materials, polymerization‐induced microphase separation, reversible addition–fragmentation chain transfer polymerization

## Abstract

Ion‐exchange materials are commonly composed of polyelectrolyte networks in which crosslinking preserves macroscopic geometry and prevents dissolution in aqueous conditions. However, crosslinking inherently inhibits efficient swelling and mass transfer during ion‐exchange processes. Herein, a one‐step polymerization‐induced microphase separation (PIMS) approach directly using water and linear polystyrene sulfonate macromolecular chain transfer agents (macroCTAs) is developed to engineer bicontinuous nanostructured materials with rapid ion‐exchange capabilities. These materials feature water‐swollen liquid‐like polyelectrolyte domain embedded in a rigid polymer network, where the domain spacing, as determined by small angle X‐ray scattering experiments, is precisely modulated between 15 and 89 nm based on the molecular weight of the macroCTA used. As the bicontinuous nanostructure enables rapid mass transfer throughout the material bulk, the 3D printed PIMS materials are able to rapidly remove model charged dyes from solution, exhibiting a mass transfer coefficient ≈35 times higher than commercially available counterparts. This work is the first example demonstrating direct self‐assembly of water into continuous nanochannels in a well‐controlled manner as supported by time‐resolved small‐angle neutron scattering experiments during polymerization. Moreover, these nanostructured materials are readily produced using commercially available 3D printers, enabling unparalleled high‐resolution fabrication of targeted complex structures, including accurately controllable macroporous geometries and surface areas.

## Graphical abstract

Rapid ion exchange is achieved by 3D printed nanostructured polyelectrolyte materials, which significantly outperform commercially available counterparts. The nanostructures evolve through a block copolymer microphase separation mechanism, where non‐crosslinked polyelectrolyte blocks self‐assemble into a continuous independent liquid‐like domain during polymerization, which facilitates rapid mass transfer throughout the material bulk.

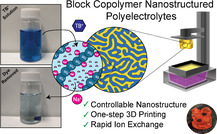

## 1 Introduction

Ion‐exchange resins are crosslinked polymeric materials containing ionic repeating units with fixed charge density that is conveniently tunable through synthesis parameters. These materials are particularly useful as they combine desirable polymer properties, such as thermomechanical and chemical stability, with highly efficient and selective ion exchange,^[^ ^1^ ^]^ which have proven invaluable in applications such as separations,^[^ ^2^ ^]^ chemical synthesis and purification,^[^ ^3^ ^]^ wastewater treatment,^[^ ^4^, ^5^ ^]^ biomedical applications,^[^ ^6^ ^]^ water purification,^[^ ^7^, ^8^ ^]^ catalysis,^[^ ^9^ ^]^ and electrodialysis.^[^ ^10^, ^11^ ^]^ For practical use, an ion‐exchange resin must be insoluble in aqueous media, which enables efficient separation by filtration, facile incorporation in continuous processes, and excellent recyclability.^[^ ^1^ ^]^ To achieve aqueous insolubility, polyelectrolytes may be covalently crosslinked, permanently fixing the material geometry, or modified to include hydrophobic regions into the polymer. However, these approaches often cause the material to exhibit unfavorable swelling behavior and slower overall mass transfer, resulting in a competition between optimizing the material for polyelectrolyte insolubility and efficient ion exchange.^[^ ^12^ ^]^

An elegant solution to this competition is the use of polymerization‐induced microphase separation (PIMS),^[^ ^13^, ^14^, ^15^ ^]^ which produces continuous, nanoscale, non‐crosslinked polyelectrolyte domains embedded within a water‐insoluble, crosslinked polymer network. In the PIMS process, a macromolecular chain transfer agent (macroCTA) is chain extended with a monomer and crosslinker, forming (macroCTA)‐*b*‐P(monomer‐*stat*‐crosslinker) diblock copolymers. With sufficient incompatibility between blocks, these diblock copolymers will begin to microphase separate. As polymerization progresses, simultaneous crosslinking arrests the emergent nanostructure, and the resulting morphology is typically bicontinuous nanostructure with two distinct domains: a linear liquid‐like macroCTA domain and a rigid crosslinked polymer network. Crucially, as these domains are formed from the self‐assembly of block copolymers, the size of each domain can be precisely controlled by modulating the size of each block, with the nanostructure readily observed using small‐angle X‐ray scattering (SAXS) or small‐angle neutron scattering (SANS) techniques. However, in all previous studies utilizing PIMS to create nanostructures with accessible charged groups, the formation of polyelectrolyte domains has followed a two‐step procedure, either through or postmodification of surface groups in the monomer‐*stat*‐crosslinker domain after material synthesis,^[^ ^16^, ^17^ ^]^ or by using precursor macroCTAs followed by postmodification to transform them into polyelectrolytes.^[^ ^12^, ^18^ ^]^ This sequential approach is necessitated by the limited solubility of charged polymers in the resin systems typically used for PIMS.

Aside from issues associated with polyelectrolyte crosslinking, ion‐exchange materials used in conventional columns are typically manufactured from randomly packed beds of spherical particles or monolithic networks. In these materials, internal morphology and pore structures may vary significantly, which necessitates additional quality management to validate packing quality.^[^ ^19^ ^]^ Consequently, there has been significant academic interest in the development of 3D printed ion‐exchange materials containing charged groups that enable precision engineered macroscopic geometries.^[^ ^20^, ^21^ ^]^ However, such developments are limited by a lack of suitable 3D printable materials that can efficiently facilitate ion exchange.^[^ ^20^ ^]^ Currently, 3D printed materials for these applications typically use weak polyelectrolytes such as poly(carboxyethyl acrylate),^[^ ^22^ ^]^ or copolymerization of strongly disassociating ionomers with polyethylene glycol‐based acrylates,^[^ ^20^, ^23^ ^]^ which have limited ion‐exchange performance.

In this work, we develop a one‐step PIMS process to produce 3D printed ion‐exchange materials composed of self‐assembled water–polyelectrolyte nanochannels embedded within a crosslinked polymer matrix. To the best of our knowledge, this work demonstrates the first one‐step synthesis of a PIMS material containing a polyelectrolyte macroCTA domain, and the first example of a PIMS system where liquid water can self‐assemble into continuous domains with precise control over domain spacing and nanochannel size. In contrast to previous PIMS processes, our approach also enables the preparation of complex macroscopic objects via a commercial 3D printer.

## 2 Results and Discussion

### 2.1 Design of PIMS Systems Using Polyelectrolyte MacroCTAs

A PIMS system is composed of a homogeneous mixture of monomer, crosslinker, macroCTA, and radical initiator, chosen such that polymerization forms a (macroCTA)‐*b*‐P(monomer‐*stat*‐crosslinker) diblock copolymer that begins self‐assembly during polymerization as driven by the thermodynamic incompatibility between blocks. Notably, the requirement for a homogeneous initial mixture presents challenges for strongly disassociating polyelectrolyte macroCTAs,^[^ ^14^ ^]^ which, although possessing good solubility in water, are typically insoluble in most organic solvents (especially in sodium form) and in bulk monomer mixtures.^[^ ^24^ ^]^ Building upon the foundation of a conventional bulk PIMS system, it has been demonstrated that incorporating a solvent that is compatible with the macroCTA, but incompatible with the monomer‐*stat*‐crosslinker block, forces selective partitioning of the solvent into the macroCTA domain while retaining a well‐controlled microphase separation regime.^[^ ^25^, ^26^ ^]^ Thus, it was hypothesized that water could be used as a cosolvent to formulate an appropriate mixture that can develop bicontinuous polyelectrolyte–water nanochannels within an insoluble, crosslinked polymer network.

To achieve this, judicious selection of monomer and crosslinker was required as these components must be initially miscible with water, but develop incompatibility upon polymerization. Diethyl acrylamide (DEAm) was selected as it is miscible with water, while P(DEAm) is known to have limited aqueous solubility, exhibiting a lower critical solution temperature (LCST) of ≈32 °C.^[^ ^27^ ^]^ However, the solubility and LCST of crosslinked thermoresponsive polymers are known to differ significantly from their linear counterparts,^[^ ^28^ ^]^ and we hypothesized that forming a densely crosslinked polymer network by combining DEAm with methylene bisacrylamide (MBAm) as crosslinking comonomer would provide materials with water insolubility. To probe this effect, DEAm was photopolymerized with 20 mol% MBAm in the presence of 40, 50, and 60 weight percent (wt%) deionized water under 405 nm light for 10 min with 0.5 wt% diphenyl(2,4,6‐trimethylbenzoyl)phosphine oxide (TPO), a photoinitiator commonly used in digital light processing (DLP) 3D printing.^[^ ^29^, ^30^, ^31^ ^]^ While initial solutions were homogeneous, the resulting polymer matrix was fully opaque (Figure S1, Supporting Information), indicating strong incompatibility between water and the resulting poly(DEAm‐*stat*‐MBAm) network at 20 mol% MBAm, thus causing the partitioning of water via macrophase separation into larger (diameter >200 nm) domains that scatter visible light.

Poly(styrene sulfonate) (PSS) is a strongly disassociating polyelectrolyte commonly used in commercial ion‐exchange resins.^[^ ^32^ ^]^ Additionally, sulfonate‐containing polyelectrolyte–polyacrylamide block copolymers have been shown to exhibit excellent self‐assembling properties in dilute solution, suggesting good suitability for use in a PIMS system.^[^ ^33^, ^34^, ^35^, ^36^ ^]^ Thus, to create bicontinuous PIMS materials for efficient ion exchange, a series of PSS macroCTAs (PSS‐CTA) with 10, 20, 40, 80, and 160 kDa were synthesized directly from 4‐styrenesulfonic acid sodium salt using aqueous thermal reversible addition–fragmentation chain transfer (RAFT) polymerization using 4‐((((2‐carboxyethyl)thio)carbonothioyl)thio)‐4‐cyanopentanoic acid as the RAFT agent (reagent components available in Table S1, Supporting Information).^[^ ^37^, ^38^, ^39^ ^]^ Successful preparation of the PSS‐CTAs was confirmed by ^1^H nuclear magnetic resonance (NMR) spectroscopy (Figure S2–S5, Table S2, Supporting Information) through the disappearance of peaks associated with vinyl bond protons and size exclusion chromatography (Figure S6 and S7, Table S2, Supporting Information).

**Figure 1:**
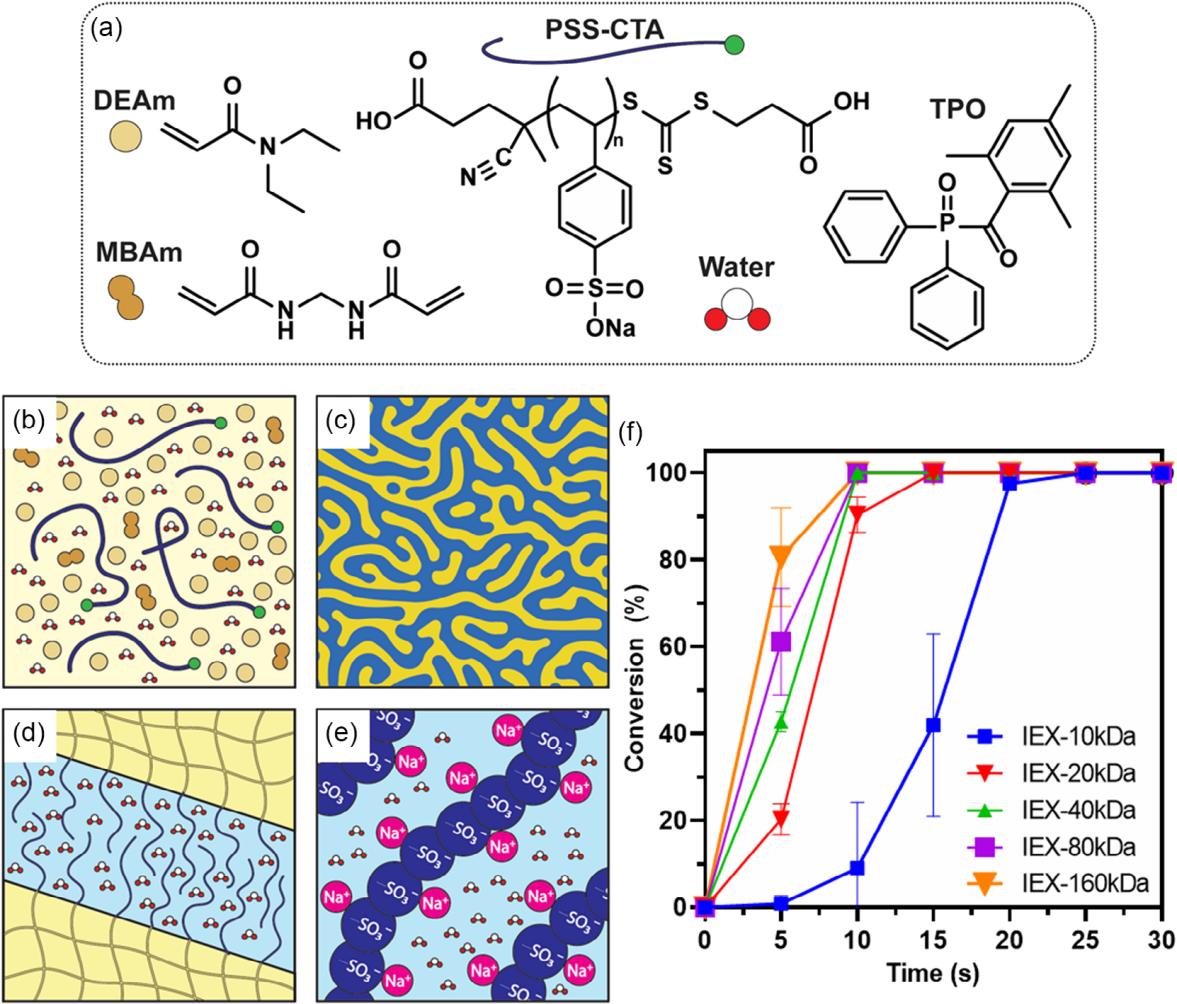
Schematic of a) resin components; b) initial solution of PSS‐CTA, MBAm, DEAm, and water; c) bicontinuous structure formed by the microphase separation of PSS‐*b*‐P(DEAm‐*stat*‐MBAm); d) separation of components where the PSS and water are isolated in polyelectrolyte nanochannels embedded within a crosslinked polymer matrix; e) illustration of the water/polyelectrolyte domain showing the presence of PSS groups which can facilitate cation‐exchange processes; and f) photopolymerization kinetics of each PIMS formulation using different molecular weight PSS‐CTAs as determined by ATR‐FTIR by monitoring the disappearance of the vinyl bond bending mode peak at 955–1000 cm^−1^. Lines through datapoints are a guide to the eye.

To formulate a PIMS system with PSS‐CTA, PSS‐CTA was combined with DEAm and MBAm ([DEAm]:[MBAm] = 4:1) and water. Initially, the water content was varied from 40 to 60 wt%, and 55 wt% water was determined to be the minimum quantity required to form a homogeneous solution that was stable at room temperature. Importantly, reducing the quantity of water was desirable to increase the solid content, which provides mechanical stability and good printability. Additionally, a higher solid content increases the amount of active PSS‐CTA and sodium sulfonate groups that facilitate efficient ion exchange. Thus, a formulation using the least water required to form a homogeneous solution was chosen, with a total solution consisting of 55 wt% water, 27.3 wt% DEAm, 8.3 wt% MBAm, 8.9 wt% PSS‐CTA, and 0.5 wt% TPO (**Figure** **1**a). Formulations featuring PSS with different molecular weights are labeled as IEX‐yyykDa, where yyy is the molecular weight of the PSS‐CTA. Following photopolymerization of the IEX‐20 kDa formulation under 405 nm light for 10 min, the resulting material was fully translucent, in contrast to materials containing only DEAm, MBAm, and water without PSS‐CTA, which were fully opaque. This difference in opacity supports a dominant microphase separation mechanism (Figure 1b–e) facilitated by the chain extension of PSS‐CTA, where nanoscale self‐assembled domains are smaller than required to scatter visible light.

### 2.2 Investigating Photopolymerization Kinetics and Optimizing 3D Printing Conditions

To determine the suitability of the PIMS materials for use in DLP printing, the photopolymerization of each formulation was followed over time using attenuated total reflectance‐Fourier transform infrared spectroscopy (ATR‐FTIR) (Figure S8, Supporting Information). Here, 20 μL of each formulation was irradiated for 30 s (intensity *I*_*0*_ = 2.06 mW cm^−2^) under open‐air conditions, and the conversion was observed by monitoring the disappearance of the vinyl bond bending mode peak (955–1000 cm^−1^). All formulations showed extremely rapid polymerization, achieving high conversions *α* > 80% within 20 s (Figure 1f). The polymerization was markedly slower with IEX‐10 kDa, whereas samples with higher molecular weight PSS‐CTA were proportionally faster. This behavior was attributed to the absorption of 405 nm light by the thiocarbonyl group in the PSS‐CTA, which reduced the amount of light available to activate the photoinitiator (TPO).^[^ ^40^ ^]^ The higher ratio of thiocarbonylthio groups to TPO in lower molecular weight PSS‐CTAs results in less TPO photolysis due to competitive light absorption.^[^ ^41^, ^42^, ^43^ ^]^ In addition, the slight increase of viscosity in the resins containing higher molecular weight PSS may also have contributed to an increased polymerization rate. Nonetheless, the rapid polymerization observed for all formulations demonstrates good suitability for DLP 3D printing.

**Figure 2:**
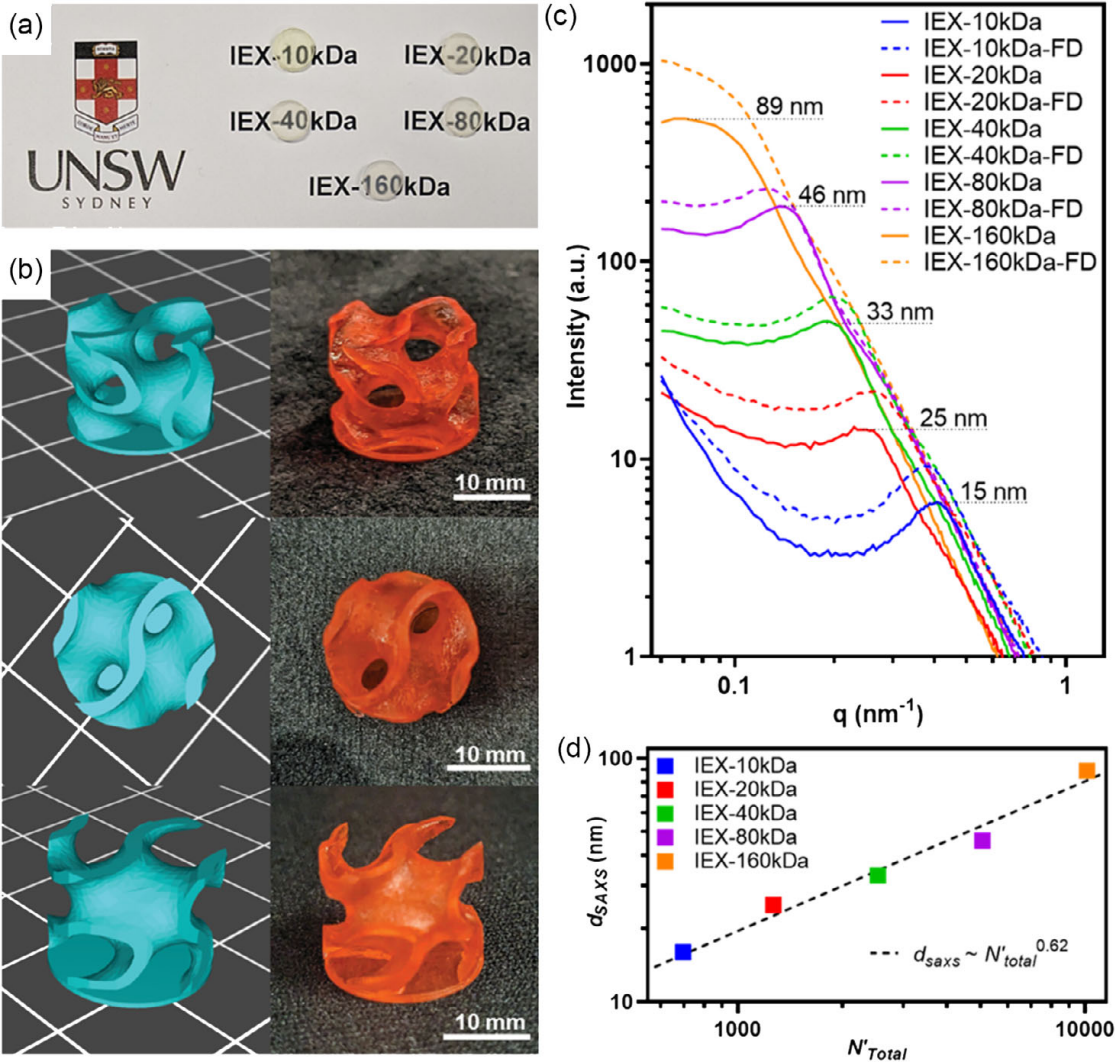
Characterization of 3D printed PIMS materials containing polyelectrolyte/water nanochannels prepared using PSS macroCTAs with a molecular weight ranging from 10 to 160 kDa. a) Photograph of translucent IEX formulation 3D printed model discs (diameter × thickness = 8 mm × 1 mm) with varying PSS‐CTA molecular weight; b) example cylindrical gyroid (12 mm height, 15 mm diameter) using 0.01 wt% Sudan II photoabsorber 3D models (left) and corresponding photographs (right); c) SAXS data for as‐printed PIMS materials before and after freeze‐drying (‐FD); and d) domain spacing derived from SAXS as a function of normalized total degree of polymerization (*N*’_total_). Dashed line indicates power law fit with *R* ^2^ = 0.97.

Each formulation was printed using a commercial DLP 3D printer with a violet light‐emitting diode array using a 50 μm slicing thickness to prepare thin discs (*d* × *t* = 8 mm × 1 mm) as model 3D printed objects (**Figure** **2**a). For all IEX materials, the resultant 3D printed discs were faithful geometric reproductions of the 3D model and reproducibly retained the targeted thickness and diameter within ±0.05 mm, indicating good suitability of using 50 μm slicing thickness. Each formulation was readily printable into well‐defined translucent objects, with slightly varied cure time between 14 and 40 s to account for the disparity in polymerization rate between formulations (cure times are given in Table S3, Supporting Information). When printing more complex objects, poor resolution was observed when forming negative features where scattered light can readily cause overcuring. This is a well‐known problem in DLP photopolymerization, which typically necessitates the use of a photoabsorber to improve print resolution in all axes.^[^ ^41^, ^43^, ^44^, ^45^, ^46^ ^]^ After the addition of 0.01 wt% Sudan II, a common photoabsorber for 405 nm light,^[^ ^47^, ^48^ ^]^ a cylindrical gyroid object was successfully printed from the IEX‐20 kDa formulation (Figure 2b), demonstrating material suitability for complex additive manufacturing.

### 2.3 Nanostructural Characterization of PIMS 3D Printed Samples

SAXS was used to investigate the 3D printed material nanostructure, where all materials exhibited a single broad SAXS peak indicative of a disordered microphase separated morphology (Figure 2c). Crucially, the characteristic domain spacing was dependent on the molecular weight of PSS‐CTA used, with spacings observed between 15 nm for IEX‐10 kDa and 89 nm for IEX‐160 kDa. From block copolymer microphase separation theory,^[^ ^49^ ^]^ a power law generally correlates domain spacing (*d*) with block copolymer size (*N*, the total degree of polymerization). A *d*≈*N* ^1/2^ relation indicates weak segregation where polymer chains contain a Gaussian distribution of conformations, whereas *d*≈*N* ^2/3^ indicates strong segregation where polymer chains are perturbed and adopt more stretched configurations. The SAXS data indicate that the characteristic domain spacing follows *d*_SAXS_≈*N* ^*’*^_total_^0.62^ (Figure 2d), which is close to *d*≈*N* ^2/3^, indicating that polymer chains adopt a more elongated configuration, likely arising from the strong incompatibility between P(DEAm‐*stat*‐MBAm) and the PSS/water domain. Ultimately, SAXS data demonstrate that the characteristic domain spacing can be tightly controlled by modulating the PSS‐CTA molecular weight in a highly predictable manner. Such bicontinuous materials with well‐controlled domain sizes are highly desirable in size‐selective separation and mass transfer applications.^[^ ^50^, ^51^ ^]^

To further characterize the nanostructured materials developed in these PIMS systems, 3D printed samples were freeze‐dried to remove water without damaging the structure, and the resulting dry materials were analyzed by SAXS. After freeze‐drying, a loss of ≈40 wt% was observed, which is slightly lower than the initial mass of water (≈55 wt%) as potentially attributed to factors including evaporation during the 3D printing and postcuring, drying during storage, reabsorption of water in the hygroscopic PSS domain from ambient conditions, or a small degree of water syneresis during polymerization. SAXS data of the freeze‐dried samples exhibited a single broad peak, indicating retention of the microphase separated nanostructure. For each IEX formulation, characteristic domain spacing was not significantly altered after freeze‐drying, likely due to the robust mechanical stability provided by the crosslinked P(DEAm‐*stat*‐MBAm) domain. Notably, SAXS data of freeze‐dried samples have higher intensity than for as‐printed materials, consistent with previous reports of PIMS‐derived mesoporous materials.^[^ ^52^, ^53^ ^]^ For water, the scattering length density (*ρ*) of water is *ρ*_Water_ = 9.4 × 10^−6^ Å^−2^, while the void space developed after freeze‐drying is *ρ*_Voids_ = 0. As intensity *I*(*q*)≈(Δ*ρ*)^2^, the increased scattering intensity observed is likely due to the increased electron density contrast between voids and the crosslinked polymer domain compared to the water–polymer domain after freeze‐drying (in the crosslinked domain *ρ*_*P(DEAm)*_  = 10.2 × 10^−6^ Å^−2^ and *ρ*_*P(MBAm)*_ = 11.0 × 10^−6 ^Å^−2^). Further supporting characterization (SEM, nitrogen sorption experiments) of all freeze‐dried materials is available in Figure S9 and S10b,c, Table S4 (Supporting Information).

In a typical PIMS system, polymerization forms macroCTA‐*b*‐P(monomer‐*stat*‐crosslinker) diblock copolymers which rapidly self‐assemble into chemically independent domains with size related to each block. Self‐assembly is kinetically impeded by simultaneous crosslinking processes, precluding the formation of globally ordered morphologies and resulting in a disordered block copolymer nanostructure. In this work, we show that the characteristic domain spacing can be controlled by the macroCTA molecular weight. However, these systems are composed of a high glass transition temperature (*T*_g_) polymer (P(DEAm) *T*_g_  = 81 °C) with high crosslinker content, and photopolymerizes extremely quickly (*α* > 80% at 20 s for IEX‐10 kDa, and at 10 s for all other samples), which may result in a stronger arrest of the nanostructure. Thus, to observe the effect of rapid photopolymerization on the evolution of PIMS nanostructures, we performed ex situ time‐resolved SANS of the self‐assembly of PSS‐*b*‐P(DEAm‐*stat*‐MBAm) during photopolymerization.

**Figure 3:**
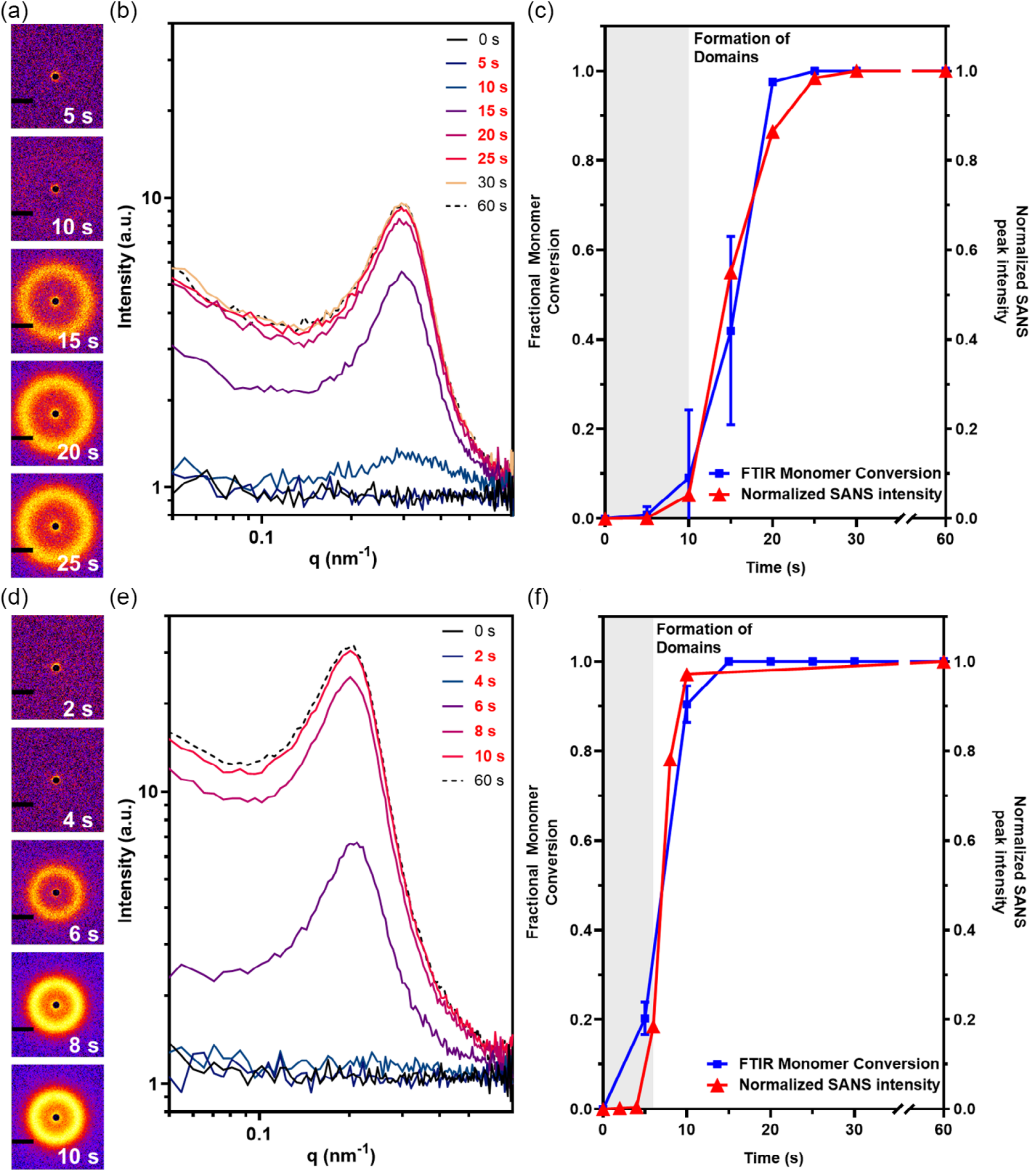
SANS data characterizing the evolution of nanostructure during the photopolymerization of IEX‐10 kDa and IEX‐20 kDa liquid resin. a) IEX‐10 kDa 2D SANS data; b) IEX‐10 kDa 1D SANS data; c) comparison of normalized 1D SANS peak intensity (normalized based on peak intensity at 60 s irradiation) with FTIR‐derived fractional monomer conversion for IEX‐10 kDa; d) IEX‐20 kDa 2D SANS data; e) IEX‐20 kDa 1D SANS data; and f) comparison of normalized 1D SANS peak intensity (normalized based on peak intensity at 60 s irradiation) with FTIR‐derived fractional monomer conversion for IEX‐20 kDa. 2D SANS data in (a) and (d) have corresponding 1D SANS data in (b) and (e), respectively, as identified in the legend in red. Lines through datapoints are a guide to the eye.

The polymerization resin (300 μL) used for 3D printing (IEX‐10 kDa, **Figure** **3**a–c, and IEX‐20 kDa, Figure 3d–f) was introduced into a demountable cell and SANS data collected, after which the cell was removed, irradiated through the quartz windows with the same 405 nm light used for the photopolymerization kinetics experiments (*I*_*0*_ = 2.06 mW cm^−2^), and SANS data were recollected. Initially, no correlation peak was observed for IEX‐10 kDa (5 s) or IEX‐20 kDa (2 and 4 s), with these irradiation periods yielding only low double bond conversion (*α* < 10%, Figure 3c,f). At this double bond conversion, the total degree of polymerization (*N*) in the P(DEAm‐*stat*‐MBAm) block is low, which may not exhibit sufficient segregation strength (*χN*) to promote phase separation. A correlation peak corresponding to double bond conversion of 15%–30% with similar *q* value to that observed in SAXS data appeared for both samples, by 10 s for IEX‐10 kDa and 6 s for IEX‐20 kDa, with intensity that increased as the polymerization proceeded. The development of the correlation peak suggests strong incompatibility between segments such that only a modest degree of polymerization is required to achieve a *χN* segregation strength capable of facilitating microphase separation. Crucially, at all stages of polymerization, the peak position indicates a consistent domain spacing, in agreement with scattering data previously reported thermal PIMS systems.^[^ ^54^ ^]^ In summary, although the emergence of an ordered structure is inhibited by crosslinking and gelation effects, the characteristic domain spacing of the disordered nanostructure does not change during polymerization and is thus predominantly defined by the macroCTA molecular weight.

The PSS‐CTA and water content were further varied, and after 3D printing the effect on nanostructure was investigated using SAXS. Using 20 kDa PSS‐CTA as a model system, the PSS‐CTA content was raised from 20 to 30 wt% relative to DEAm and MBAm (8.9–14.7 wt% overall) while retaining 55 wt% water. Although homogeneous solutions formed, well‐defined 3D printed objects were not able to be prepared. Increasing the water to 60 and 70 wt%, while retaining 8.9 wt% 20 kDa PSS‐CTA, enabled successful 3D printing, with materials remaining translucent. However, SAXS data indicated a less well‐defined microphase separated nanostructure (Figure S10a, Supporting Information) indicated by a reduction in peak intensity and narrowness. Hence, samples containing 8.9 total wt% PSS‐CTA and 55 wt% water were used for all further experiments.

### 2.4 Ion‐Exchange and Dye‐Sorption Capabilities of 3D Printed PIMS Materials

A significant advantage of the PIMS bicontinuous nanostructure for ion‐exchange applications is the formation of continuous nanochannels that harbor liquid‐like linear polyelectrolyte chains that can rapidly facilitate mass transfer. The ion‐exchange capacity (IEC) was measured to determine the number of charges present in the 3D printed material by titration using established protocols.^[^ ^12^, ^17^ ^]^ Initially, sodium ions in the PSS‐CTA domain of 3D printed materials were substituted for protons through immersion in 0.1 M H_2_SO_4_, followed by thorough washing and immersion in deionized water, and then an additional ion exchange accomplished by soaking in 1 M NaCl. The residual HCl produced by the second ion‐exchange process was back titrated against 0.01 M NaOH to provide a measurement of IEC. The IEC values (**Table** **1**) were consistently 0.36–0.38 mmol g^−1^ for all samples, similar to that expected based on the initial resin components (≈82.7–87.2% of theoretical IEC). This evidences the formation of continuous channels that are largely able to facilitate the mass transfer of ions throughout the material. The remaining ≈15% loss in IEC was attributed to the presence of unreactive PSS homopolymers from the original PSS‐CTA synthesis which would be leached out in soaking processes, potential errors in titration measurement given the relatively low loading of material (≈55 mg), or the unavoidable formation of minor isolated PSS domains which cannot be reached from the continuous domain in the final material.

**Table 1:** Characterization of 3D printed PIMS ion‐exchange materials containing liquid‐like polyelectrolyte/water nanodomains

Name	*d*_SAXS_	Water loss [wt%]	IEC _titration_ [mmol g^−1^]	Percent of theoretical IEC [%]	Mass transfer coefficient, *k* [s^−1^]
IEX‐10 kDa	15	38.8	0.37 ± 0.01	83.8 ± 1.4	0.00011
IEX‐20 kDa	25	42.0	0.36 ± 0.01	82.8 ± 2.6	0.014
IEX‐40 kDa	33	41.0	0.37 ± 0.01	83.9 ± 2.2	0.010
IEX‐80 kDa	46	43.5	0.38 ± 0.01	86.2 ± 1.6	0.0050
IEX‐160 kDa	89	40.8	0.36 ± 0.01	82.7 ± 1.8	0.0099
Amberlite IRC120 Na	–	–	–	–	0.00028

**Figure 4:**
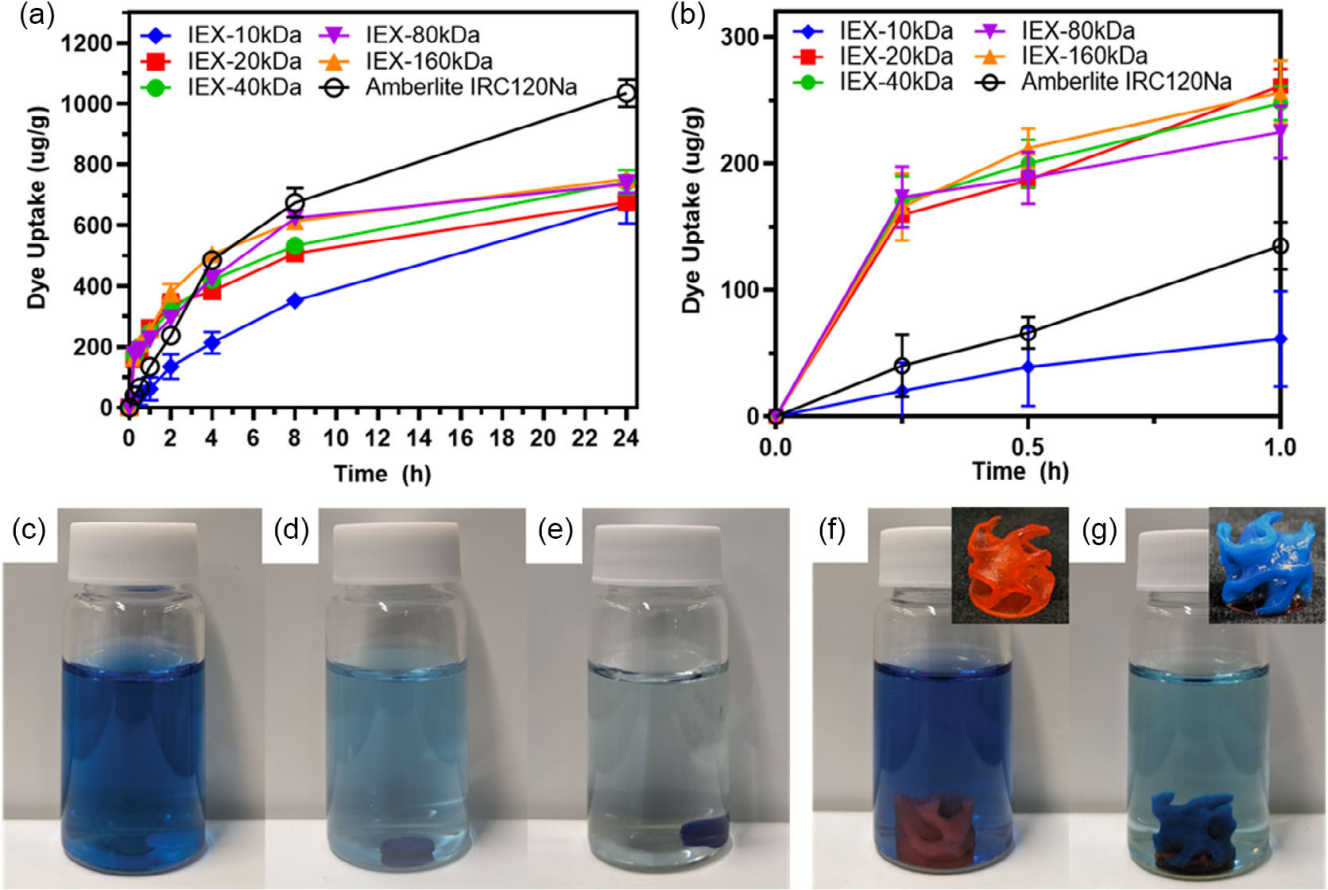
Dye uptake per mass as measured using TB in phosphate‐buffered solution during the a) first 1 h; b) first 24 h; photograph of the IEX‐20 kDa sample submerged in dye solution at c) 0 h, d) 24 h, e) 8 weeks; IEX‐80 kDa cylindrical gyroid shape submerged in TB solution after f) 0 h and g) 24 h; inset images depict 3D printed gyroid before and after submersion. Lines through datapoints are a guide to the eye.

An 8 mm × 1 mm 3D printed disc of each formulation was submerged for 24 h in a temperature‐controlled orbital shaker in 15 mL of phosphate‐buffered toluidine blue (TB, 0.5 mg/100 mL), a cationic dye expected to participate in ion exchange with PSS‐Na (**Figure** **4**). The rate of dye ion exchange was measured by monitoring the concentration of residual dye in solution over time via UV–vis spectroscopy through absorbance at 635 nm (Figure S12, Supporting Information). A commercial PSSNa‐based strong acid cation‐exchange material, Amberlite IRC 120 Na, was also tested under the same conditions using a similar mass loading (≈60 mg).^[^ ^32^ ^]^ Data were fitted using a power law model (Korsmeyer‐Peppas)^[^ ^55^ ^]^ to determine the mass transfer coefficient *k* (Figure S13, Table S5, Supporting Information).

For all formulations except IEX‐10 kDa, extremely rapid dye uptake was observed in the first 15 min, followed by a gradual plateauing (Figure 4a). Excluding IEX‐10 kDa, the measured absorbance at each interval was typically within error of repeats, with no significant difference between samples, as reflected in the calculated mass transfer coefficient (Table 1, *k* = 0.01 s^−1^ for IEX‐20 kDa, IEX‐40 kDa, IEX‐80 kDa, and IEX‐160 kDa). For IEX‐10 kDa, slower dye uptake was observed in the first 8 h, as reflected in the smaller mass transfer coefficient (*k* = 0.00011 s^−1^). Despite having an initially slower dye absorption, IEX‐10 kDa absorbed approximately the same quantity of dye after 24 h as all other samples (≈650 μg g^−1^, 60% total dye removal, Figure 4b). This plateau point is expected from the experimental IEC, where all samples had the same IEC after extensive equilibration and back titration. After approximately 8 weeks of submerging in dye solution, some additional absorption occurred beyond the 24 h plateau point for IEX‐20 kDa (1183 μg g^−1^ TB removal, 94% total dye removal, Figure 4e and S14, Supporting Information).

The amount of dye absorbed by the commercial Amberlite IRC120 Na resin after 24 h was significantly higher than for all 3D printed PIMS materials (1035 μg g^−1^ for Amberlite IRC 120 Na compared to 650 μg g^−1^ for 3D printed resins). This behavior is expected, considering that only ≈10 wt% of the PIMS material is PSS, compared with the commercial ion‐exchange resin that is predominantly composed of charged polymers. Aside from IEX‐10 kDa, all samples exhibited a significantly higher rate of dye absorption in the first 2 h than the commercial Amberlite IRC 120 Na, absorbing up to 3 times as much dye within 30 min. This is reflected in the mass transfer coefficient, which is ≈35 times higher for the 3D printed PIMS materials (*k* = 0.01 s^−1^) than Amberlite IRC 120 Na (*k* = 0.0003 s^−1^). Notably, these results are obtained for discs with a relatively low surface area to volume ratio compared with the small bead format of Amberlite IRC 120 Na. The higher rate of dye uptake observed for the 3D printed materials is attributed to the swelled liquid‐like water/polyelectrolyte domains created by PIMS, where dye molecules can diffuse into the material much faster than in crosslinked polyelectrolyte ion‐exchange resins. The significantly more rapid ion exchange of the 3D printed materials may prove more useful than commercial analogues in flow applications with short residence times, or where relatively low concentrations of impurities need to be efficiently removed. In addition to the disc shapes used in the original dye experiments, the cylindrical gyroid shape displayed in Figure 2d was 3D printed using the IEX‐80 kDa formulation and tested for dye absorption capabilities. Pleasingly, the gyroid object was able to remove 80% of total TB content from the original solution in 24 h (Figure 4f–g and S15, Supporting Information), demonstrating the suitability of the PIMS 3D printing process for the fabrication of ion‐exchange materials in arbitrary complex geometries.

## 3 Conclusion

Ion‐exchange materials with a bicontinuous nanostructured water/polyelectrolyte domain embedded into a crosslinked polymer network were successfully produced via a single‐step photo‐polymerization‐induced microphase separation (photo‐PIMS) 3D printing process. Here, the PIMS process facilitates the formation of two continuous yet chemically distinct nanodomains: a water‐swelled non‐crosslinked polyelectrolyte domain that facilitates rapid mass transfer throughout the material, and a rigid crosslinked polymer network that provides robust mechanical integrity required for 3D printing. Notably, digital light processing 3D printing of these systems allowed the manufacturing of accurate and complex macroscale geometries which are difficult to achieve with conventional polyelectrolyte materials. As ion‐exchange materials, the preswelled non‐crosslinked PSS chains in the macroCTA domain rapidly exchanged with free ions in solution, and were able to remove a model charged cationic dye from solution ≈35 times faster than a commercial ion‐exchange material. As an additional novelty, the PIMS system explored in this work also effectively forces water to partition into highly tunable nanodomains via self‐assembly, with a domain size that is readily tuned between 15 and 89 nm through modulation of the PSS‐CTA molecular weight. In addition to ion‐exchange applications explored in this work, such new materials may prove useful in drug delivery, aqueous electrochemical devices, catalysis, electrodialysis, or other biomedical applications.

## Conflict of Interest

The authors declare no conflict of interest.

## Supporting information

Supplementary Material

## Data Availability

The data that support the findings of this study are available in the supplementary material of this article.
