# Supplementary material for: 3D Printing Highly Efficient Ion‐Exchange Materials via a Polyelectrolyte Microphase Separation Strategy
Source: Small Sci. 2024 Mar 10;4(5):2400019. doi: 10.1002/smsc.202400019 (PMC11935272; doi:10.1002/smsc.202400019)
Supplement: Supplementary file 1 — Supplementary Material [file SMSC-4-2400019-s001.pdf]

Supporting Information

3D Printing Highly Efficient Ion Exchange Materials via a Polyelectrolyte Microphase Separation Strategy

Kenny Lee,[a] *Jitendra Mata*[e,c]*, Valentin A. Bobrin*[a]*, Dipan Kundu*[c,d]*, Vanessa K. Peterson*[e]*, Nathaniel Corrigan,*[a,b*] *and Cyrille Boyer*[a,b]*

[a] Cluster for Advanced Macromolecular Design (CAMD) and [b] Australian Centre for NanoMedicine (ACN), School of Chemical Engineering, UNSW Australia, Sydney, NSW 2052, Australia

[c] School of Chemical Engineering, UNSW Sydney, Kensington, NSW 2052, Australia

[d] School of Mechanical and Manufacturing Engineering, UNSW Sydney, Kensington, NSW 2052, Australia

[e] Australian Centre for Neutron Scattering (ACNS), Australian Nuclear Science and Technology Organisation (ANSTO), Lucas Heights, NSW 2234, Australia

*E-mail: cboyer@unsw.edu.au, n.corrigan@unsw.edu.au

1. Materials and Methods

1.1. Materials

Methylene bisacrylamide (MBAm, ≥ 99 %), 4-styrenesulfonic acid sodium salt (SSNa), toluidine blue O (TB, technical grade), diphenyl(2,4,6-trimethylbenzoyl)phosphine oxide (TPO, ≥ 97%), Sudan II (Dye content > 90%), trimethylolpropane triacrylate (TMPTA, > 70%) and Amberlite IRC120 Na form were purchased from Sigma Aldrich and used as received. Diethyl acrylamide (DEAm, ≥ 98%), acetone (≥ 99.7%), and methanol (≥ 99.9%) were purchased from Chemsupply Australia and used as received. 4,4'-azobis(4-cyanovaleric acid) (ACVA, ≥ 98%) was purchased from Bio-Strategy Pty Ltd and used as received. 4-((((2-carboxyethyl)thio)carbonothioyl)thio)-4-cyanopentanoic acid (CTCPA, ≥ 95%) was purchased from Boron Molecular and used as received. Phosphate buffered saline tablets were purchased from Thermo Fisher scientific and dissolved in 100 mL of deionized water to formulate phosphate buffer. D_2O was purchased from Cambridge Isotope Laboratories Inc. and used as received.

1.2. Methods

Unless stated otherwise, all experimental procedures were performed on equipment and in laboratories located at the University of New South Wales (UNSW Sydney), Science and Engineering building.

1.2.1. Synthesis of PSS-CTA

PSS-CTA 10 kDa was synthesized as follows: 30.0 g SSNa (0.14 mol, 1 equiv), 0.922 g CTCPA (3.00 mmol, 0.02 equiv), 0.168 g ACVA (0.600 mmol, 0.004 equiv), and 120 g of deionized water were added to a 250 mL round bottom flask and sonicated until homogeneous. The mixture was degassed by purging with nitrogen for 30 min, then polymerized for 24 h at 70 °C. The reaction was stopped by cooling in an ice bath and exposing to air. Polymer precipitation in acetone was then performed to purify the PSS-CTA. Initially, 10 mL of polymer solution was added to 90 mL of acetone dropwise. The mixture was then vortexed and sonicated for 2 min and allowed to settle. The supernatant was then discarded, and the polymer redissolved in 10 mL of deionized water. The precipitation process was repeated 3 times after which the precipitate was dried under constant airflow for 24 h, followed by drying at 70 °C for 4 h under vacuum, and subsequently placed in a

desiccator for at least 24 h before use. Using the same protocol, PSS-CTAs with varying molecular weight were also synthesized by modifying the CTCPA content, and keeping the [CTCPA]:[ACVA] ratio at 1:0.2.

1.2.2. Preparation of samples for 3D printing and determining reaction kinetics

Noting that the order of reagent addition affected the efficiency of dissolution, typical polymerization solutions were prepared using the following procedure: for a 20 mL solution suitable for 3D printing, 1.72 g of PSS-CTA and 10.6 mL of deionized water were combined in a 20 mL glass vial. The solution was covered in aluminum foil, vortexed for 1 min, and then sonicated for 15 min (25 °C, 400 W), repeating until a homogenous solution was obtained. Then, 5.72 mL DEAm, 1.60 g MBAm, and 99 mg TPO were added simultaneously, and similarly vortexted and sonicated until homogeneous. For kinetic experiments, identical formulations were downscaled to 1 mL solutions in a 4 mL glass vial, from which 20 µL aliquots were used for each experiment.

1.2.3. 3D printing procedures

A typical procedure for fabricating 3D printed objects is as follows: A 3D object was designed using Tinkercad 3D modelling software and the object was exported as an .stl file format. The .stl file was opened using Photon Workshop slicing software. In the slicing software, the Z lift speed was set to 0.5 mm/s, the Z retract speed was set to 6 mm/s, and the Z lift distance was set to 3 mm. The model was then sliced using Photon Workshop and copied to a flash drive for use with a masked DLP 3D printer (Anycubic Photon Mono SE) with a violet (λ_{max} = 405 nm) light emitting diode (LED) array (intensity I_0 = 2 mW cm^{-2}). For the complex cylindrical gyroid object in **Figure 2b** and **Figure 4f-g**, Sudan II was added at a 0.05 wt.% loading as photoabsorber. The first 10 layers (0.5 mm) were printed using a mixture of DEAm, Sudan II, TPO and trimethylolpropane triacrylate (DEAm : TMPTA = 4 : 1, 1 wt.% TPO) with a cure time of 10 s to provide sufficient adhesion to the build plate over extended printing periods. Cure times for each formulation are provided in **Table S1**.

Upon completion of the printing program, the 3D printed objects were rinsed briefly with deionized water, and cleaned gently with lint-free wipes. While attached to the build plate, the objects were post-cured under violet light (λ_{max} = 405 nm) for 10 min. Then, objects were removed using a 0.1 mm thick stainless steel bar, which was constantly wetted with deionized water to facilitate gentle removal of the objects from the build

plate. After printing, all samples were stored in a sealed 100 mL glass jar alongside a 20 mL glass vial containing 10 mL of water in order to reduce drying.

1.2.4. Freeze-drying procedure for removal of water content

3D printed samples (approx. 200 mg of total weight from various prints) were placed into pre-weighed open 20 mL glass jars, and weighed again to measure the weight of the glass jar and the sample. The open glass jars were then fully submerged in liquid nitrogen, allowing the samples to directly contact the liquid nitrogen. After 20 min, the glass jars were removed and covered with a lint-free wipe, which was secured with an elastic band. The samples were then placed in a freeze-dryer for 48 h. After removal of the samples from the freeze-dryer, the mass of the sample was weighed to calculate the mass percentage of water removed.

1.2.5. Ion exchange capacity measurement by back-titration

1 mm thick 8 mm diameter discs were 3D printed and weighed to calculate the percentage of styrene sulfonate groups present in the 3D printed material based on the original formulation. Each disc was placed into 20 mL of 0.1 M solution of H_2SO_4 for 72 h to protonate the PSS. After thorough washing, the samples were placed into 20 mL of deionized water for a further 72 h to remove any free H_2SO_4 trapped inside the PSS/water domain. Finally, samples were submerged in 5 mL of 1 M NaCl solution where the protons in the PSS domain are exchanged with sodium ions while forming free HCl. 1 mL aliquots of solution were taken and titrated against 0.001 M NaOH solution using 20 μ L of 0.01 M phenolphthalein solution in deionized water to identify the concentration of free HCl.

1.2.6. Procedures for dye sorption experiments

Initially, a toluidine blue (TB) calibration curve (**Figure S11**) was determined by separately dissolving TB in phosphate buffer (4.55 mg/100 mL and 1.77 mg/100mL) which were then serially diluted to between 0.6 and 0.1 mg/100 mL. The calibration curve was defined by fitting the absorbance data to the Beer-Lambert law $A = \varepsilon \ell c$ where A is the absorbance, ε is the molar extinction coefficient (calculated from the calibration curve to be ε = 30518 M^{-1} cm^{-1}), ℓ is the path length (1 cm) and c is the concentration of TB.

For the preparation of TB solutions for dye uptake experiments, TB was dissolved in phosphate buffer (pH = 7.4) to 2 mg/100 mL, from which 0.5 mg/100 mL solutions were made. Each disc was initially placed in

phosphate buffer for 1 d prior to performing dye sorption experiments. Then, each disc was placed in 15 mL of the 0.5 mg/100 mL TB solutions in a 20 mL glass vial and placed in a temperature controlled orbital shaker at 25 °C and 100 rpm. Here, the 3D printed discs (8 mm diameter × 1 mm thickness) satisfy infinite slab conditions (diameter > 4 × thickness) which also have a volume more than 300 times lower than the volume of dye solution used, satisfying assumptions for near-zero concentration boundary conditions and an infinite open system.[1] A 1 mL aliquot of each solution was taken at 15 min, 30 min, 1 h, 2 h, 4 h, 8 h, and 24 h, and placed into a 1 cm quartz cuvette and analyzed by ultra violet – visible (UV-Vis) spectroscopy. The aliquots were returned to the original vials after each analysis step. For each 3D printed material, dye adsorption kinetic curves were fitted to the Korsmeyer-Peppas mass transfer model to determine mass transfer characteristics:

$$\frac{M_t}{M_\infty} = kt^n \qquad \textbf{Eq. 1}$$

Where M_t is the mass of dye adsorbed at time t, and M_∞ is the total mass of dye in solution, t is time interval at measurement, k is the mass transfer coefficient, and n is the mass transfer exponent. Each material was tested under dye sorption experiments in triplicate.

For IEX-80kDa cylindrical gyroid dye absorption experiment, the 3D printed gyroid was placed in 15 mL of 0.5 mg/100 mL TB solution and placed in a temperature controlled orbital shaker at 25 °C and 30 rpm for 24 h.

2. Characterization

2.1. ATR-FTIR spectroscopy

Attenuated total reflectance-Fourier transform infrared spectroscopy (ATR-FTIR) was performed using a Bruker Alpha FTIR spectrometer equipped with room temperature deuterated triglycine sulfate detector detectors. After taking a background reading of an empty plate, 20 μL of polymerization resin was pipetted onto the ATR crystal plate. An absorption spectrum was then created by scanning the droplet across a 400-4000 cm^{-1} range. The peak at 955-1000 cm^{-1} representing the bending mode of the vinyl C=C group was monitored as these groups are consumed as polymerization occurs. A peak at 2600-3800 cm^{-1} representing the O-H stretching mode in water was monitored as a control as this remains unchanged during polymerization.

After an initial reading, the droplet was irradiated with a Thorlabs mounted light emitting diode (LED) with a collimation adapter (λ_{max} = 405 nm, I_0 = 2.06 mW.cm^{-2}) for 30 s and subsequently scanned to create another absorption spectra. Conversion was then calculated using Equation 2.

$$Conversion\ (\%) = 100\ \times \left(1 - \frac{int_x/std_x}{int_0/std_0}\right) \qquad \textbf{Eq. 2}$$

Where int_x is the integral of the 955-1000 cm^{-1} peak and std_x is the integral of the 2600-3800 cm^{-1} peak at *x* min of irradiation, and where int_0 is the integral of the 955-1000 cm^{-1} peak and std_0 the integral of the 2600-3800 cm^{-1} peak before irradiation. The conversion was monitored at 5 s intervals for 30 s. All measurements were performed in triplicate.

2.2. Small-angle X-ray scattering (SAXS)

An Anton Paar SAXSPoint 2.0 system equipped with a Cu Kα (λ = 0.154 nm) X-ray source and Dectris Eiger 1M detector located at Sydney Analytical at the University of Sydney was used to perform SAXS experiments. A sample-to-detector distance of 0.575 m was used and experiments performed at room temperature under vacuum. 3D printed 8 mm diameter circular samples with 0.5 mm thickness were used for all SAXS experiments which were placed in a 4 × 5 20 position holder, where one position is used for a direct beam measurement. Each sample was scanned for a 5 min interval. Data were collected using Anton Paar SAXSDrive software and reduced to 1D by radial averaging the 2D detector after converting pixel positions via $q = (4\pi/\lambda)\sin\theta$, where 2θ is the scattering angle using Anton Paar SAXS Analysis software, which was also used to reduce data to account for sample thickness, transmission values, perform solid angle correction, calibrate intensity using direct beam measurements.

The power law relationship between d_{SAXS} and the total volumetric degree of polymerization N$'$ was calculated using polymer densities and normalized against a standard reference volume. The volumetric degree of polymerization for each polymer component (PSS, P(DEAm) and P(MBAm)) was calculated using on Eq. 3:

$$N' = \frac{M_{\mathrm{n}}}{\rho v_0 N_A} \qquad \textbf{Eq. 3}$$

Where for each component, $N^{'}$ is the volumetric degree of polymerization normalized by the common reference volume $v_0 = 118$ Å^3, M_n is the number average molecular weight, ρ is the polymer density, and N_A is Avogadro's number. The total degree of polymerization was then calculated by $N'_{total} = N'_{PSS} + N'_{P(DEAm)} + N'_{P(MBAm)}$. The densities for each polymer component were taken as $\rho_{PSS} = 1.163$,[2] $\rho_{P(DEAm)} = 1.072$,[3] and $\rho_{MBAm} = 1.216$ taken as the monomer density, noting that the monomer is a solid at ambient conditions and assuming negligible density change upon polymerization.

Scattering length density calculations were performed in SASView software, using the above polymer densities.

2.3. Nuclear magnetic resonance (NMR) spectroscopy

NMR samples were prepared by dissolving ~10 mg of solid sample in 600 µL of D_2O was then transferred into a 5 mm diameter NMR tube. A Bruker Avance III 300 MHz spectrometer located in the NMR facility at the Mark Wainwright Analytical Centre at the University of New South Wales was used to record ^{1}H NMR spectra taking 32 scans per sample, using D_2O as the deuterated solvent. Data was reported as a chemical shift (δ) measured in ppm downfield from tetramethyl silane. NMR data, including integral values, were analyzed using Bruker Topspin 3.6.5 analysis software.

2.4. Size exclusion chromatography (SEC)

Aqueous SEC was performed in 10 vol.% methanol in 0.2 M $NaNO_3$ and 0.07 M NaH_2PO_4 buffer (resulting in a solution of pH = 9). The polystyrene sulfonate macroCTAs were analyzed using a modular Shimadzu SEC High Pressure Liquid Chromatography equipped with a DGU-20A degassing unit, a LC-20AD solvent delivery module at a flow rate of 1 mL/min (pressure range 1000-1500 psi), an SIL-20A auto sampler, a CTO-20AC column oven operating at 25 °C, an Agilent 8.0 µm bead size guard column followed by two Agilent PL Aquagel-OH Mixed-M columns (300 mm length × 7.5 mm internal diameter, 8 µm particle size with size exclusion limits 500 Da to 500 000 Da), an RID-20A refractive index (RI) detector and a CBM-20A communication bus module. All SEC data were analyzed considering a calibration curve using retention time data for Phenomenex polystyrene sulfonate calibration standards (1670, 6430, 10 200, 33 500, 65 400, 158 000, and 305 000 g/mol, $Đ < 1.2$ for all calibration standards). A third order polynomial was used to fit the

resulting log$M$ vs retention time (RT) calibration curve, which took the form: $\log M = -0.01052672(RT)^3 + 0.5212074(RT)^2 - 8.990266(RT) + 57.77548$, where M is the molecular weight. All SEC samples including calibration standards were prepared by dissolving polymer samples in the described SEC buffer at a concentration of 4 mg mL^{-1}.

2.5. Scanning electron microscopy (SEM)

All SEM experiments, including coating procedures, were performed on equipment located in the Electron Microscope Unit at the Mark Wainwright Analytical Centre at the University of New South Wales. 5 mm x 5 mm x 5 mm cube samples were 3D printed and freeze-dried as described in 1.2.4. Samples were set on a stub using conductive tape and coated with platinum with 30 nm thickness using a Leica ACE600 sputter coater. SEM images were obtained using a field-emission NanoSEM 230 instrument with a 5 kV accelerating voltage and a secondary electron detector.

2.6. Nitrogen sorption experiments

Surface area was determined from N_2 (77 K) adsorption-desorption isotherms measured using a NOVAtouch LX^2 gas sorption analyzer. Samples were loaded in type B long non-elutriating cell kit, 9 mm large bulb with dimple and degassed for 10 h at 80 °C before measurement. The surface area was calculated using a Brunauer-Emmett-Teller (BET) method.

2.7. Ultraviolet-visible (UV-Vis) spectroscopy

A Varian Cary 300 spectrophotometer was used to record UV-Vis spectra. A scanning range of 800 – 400 nm was used with a scanning rate of 600 nm min^{-1}. All scans were performed at room temperature (22 °C) and baseline corrected using the absorbance at 800 nm. Measurements were taken by placing 1.5 mL sample aliquots in a 1 cm square quartz cuvette. Further experimental details including the formation of a calibration curve are found in section 1.2.6.

2.8. Small angle neutron scattering (SANS)

Samples of liquid 3D printing resin for IEX-10kDa and IEX-20kDa were placed in a demountable sample holder between two 20 mm diameter quartz windows, separated with a path length of 1 mm. SANS data was obtained using the Quokka SANS instrument at the Australian Center for Neutron Scattering (ACNS) at the

Australian Nuclear Science and Technology Organization (ANSTO). Considering the region of interest and based on full *q* scan data from non-kinetic samples (data not shown here), we have selected one detector distance for all kinetic SANS measurements. Data were collected using a neutron wavelength of ~ 5 $Å^{-1}$ with 20 m collimation with a detector distance of 12 m (12.5 mm sample apature) for an exposure time of 300 s, yielding the *q* range 0.004439 – 0.0712 $Å^{-1}$. Between each 300 second scan, each sample was removed from the beam and irradiated with UV light within the demountable cell, using the same light source as in the FTIR kinetic experiments (I_0 = 2.06 $mW.cm^{-2}$), for 5 s intervals for IEX-10kDa and 2 s for IEX-20kDa. Each sample was subject to 8 total measurements including an initial (t_0) measurement, and a separate last scan was taken at 60 s total irradiation for each sample to determine the scattering profile at maximum monomer conversion. Empty cell was used as background in the same configuration. All SANS data were reduced using Igor Pro computer software loaded with Centre for Neutron Research (NCNR) SANS reduction macros to subtract the appropriate background scattering from sample data and process 2D SANS images. All 1D scattering profiles were derived using Igor Pro software by radial averaging the 2D detector after converting pixel positions to the scattering vector $q = (4\pi/\lambda)\sin\theta$, where λ is the neutron beam wavelength, and 2θ is the scattering angle. The black horizontal bar in 2D SANS profiles represent faulty regions of the detector profile which are then masked during the reduction process using Igor Pro to avoid this region. Data were corrected for detector sensitivity and appropriate background was subtracted including Blocked beam measurements.

3. Additional Data

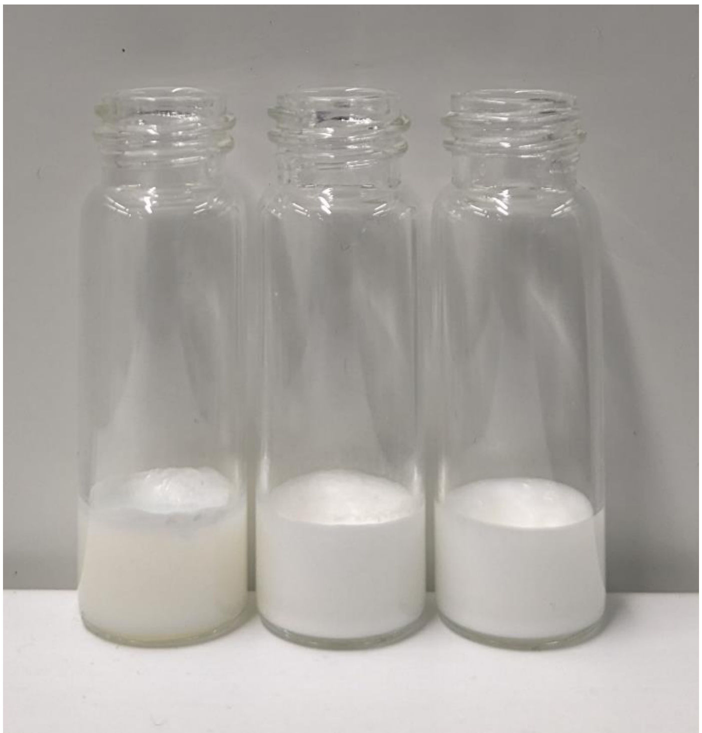

Figure S1: Photo of the polymerization mixture of water, DEAm, MBAm ([DEAm] : [MBAm] = 1 : 4) and 0.5 wt% TPO after exposure to 405 nm light for 10 min. From left to right: 40, 50, and 60 wt.% water. An opaque, white solid in all formulations indicates the partitioning of the water and polymer components into large domains that scatter visible light.

Table S1. Reagents used for the preparation of PSS-CTAs by CTCPA mediated aqueous thermal RAFT polymerization.

MacroCTA	SSNa (g)	CTCPA (mg)	ACVA (mg)	Deionized Water (mL)
10 kDa PSS-CTA	30	853.2	168.2	120
20 kDa PSS-CTA	30	426.6	84.1	120
40 kDa PSS-CTA	30	213.3	42.0	120
80 kDa PSS-CTA	30	106.7	21.02	120
160 kDa PSS-CTA	30	53.3	10.5	120

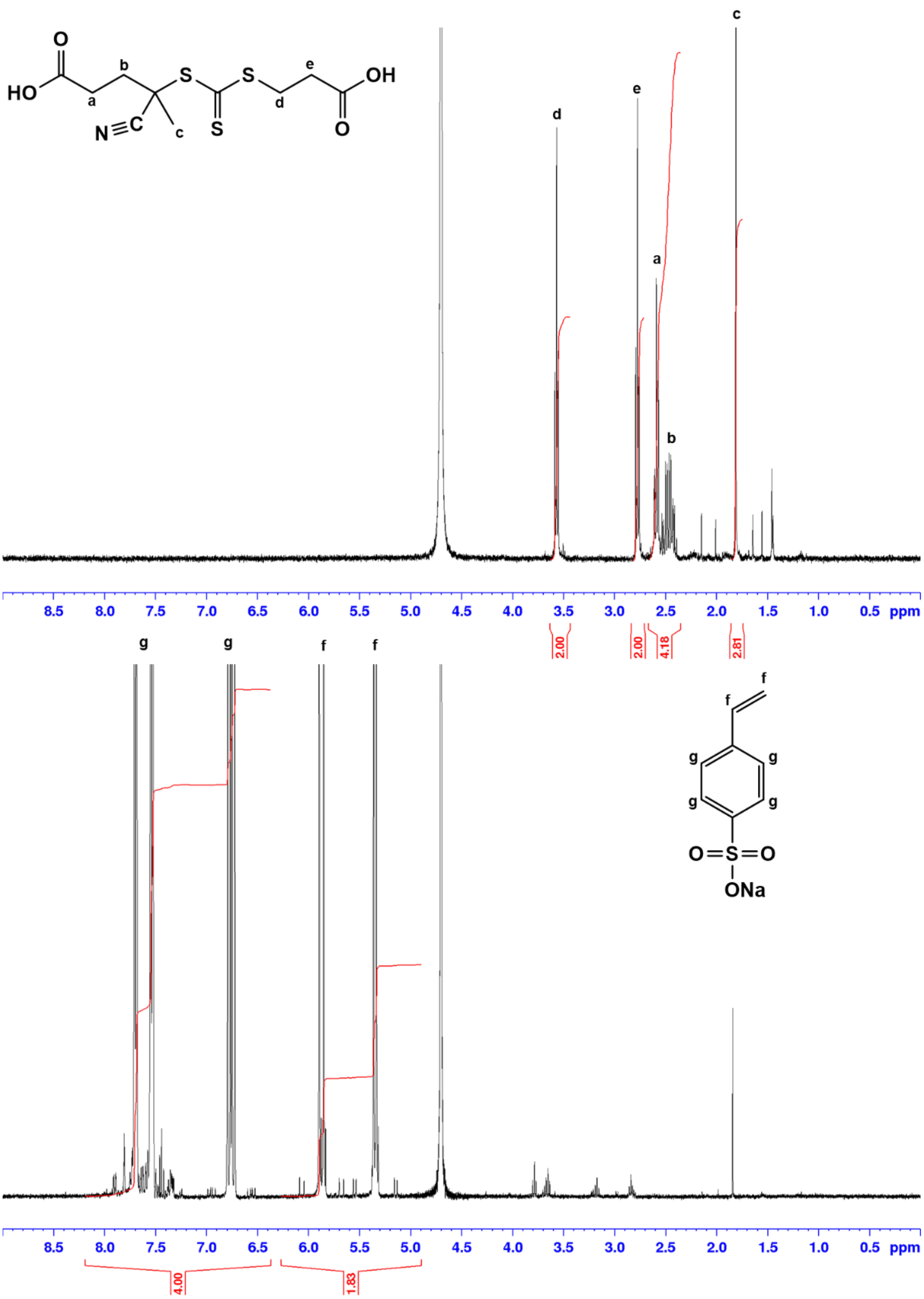

Figure S2: ^{1}H NMR spectra of CTCPA (top) and SSNa (bottom) in D_2O. Red markers indicate integrals boundaries and corresponding integral values.

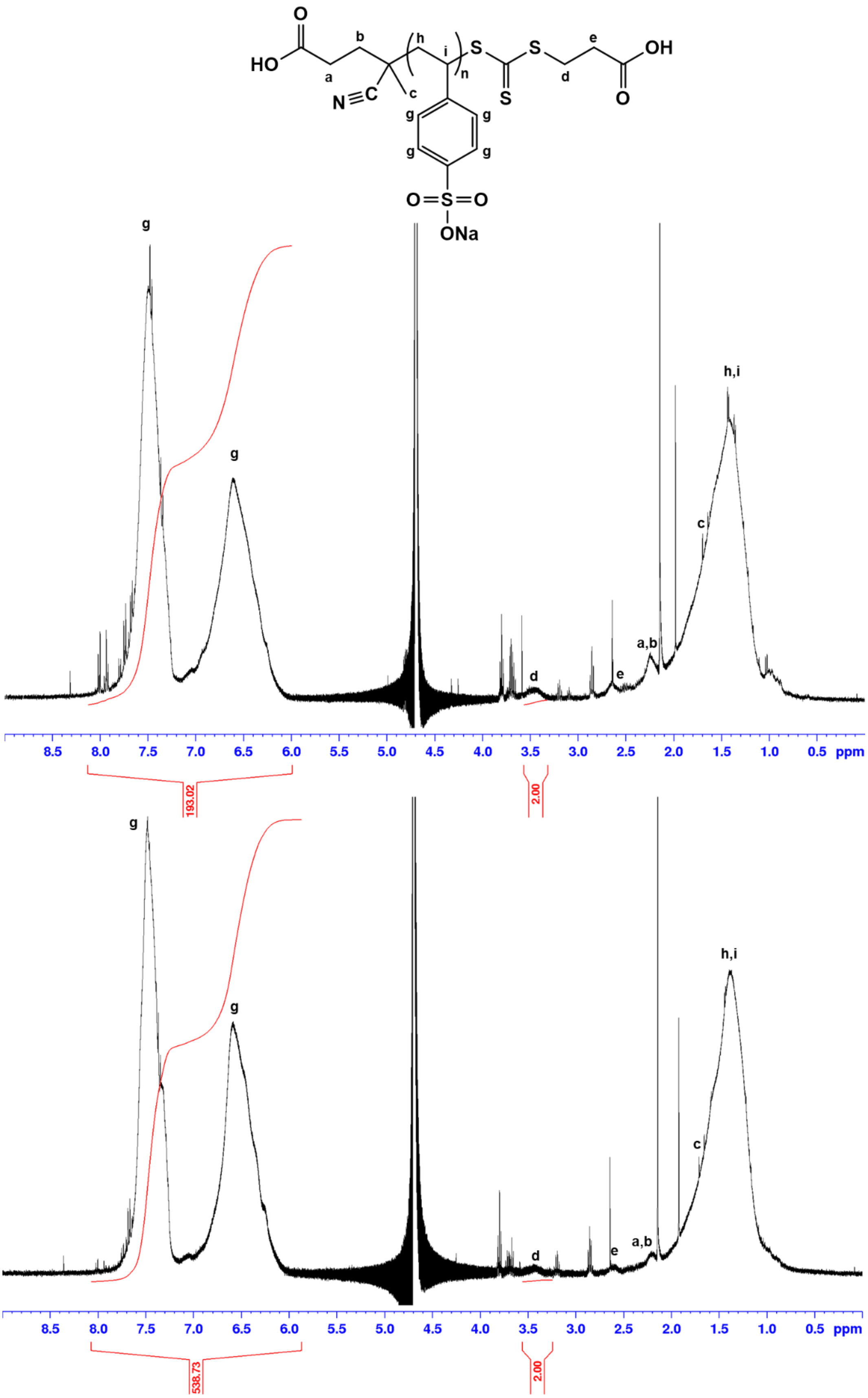

Figure S3: ^{1}H NMR spectra of 10 kDa PSS-CTA (top) and 20 kDa PSS-CTA (bottom) in D_2O. Red markers indicate integrals boundaries and corresponding integral values.

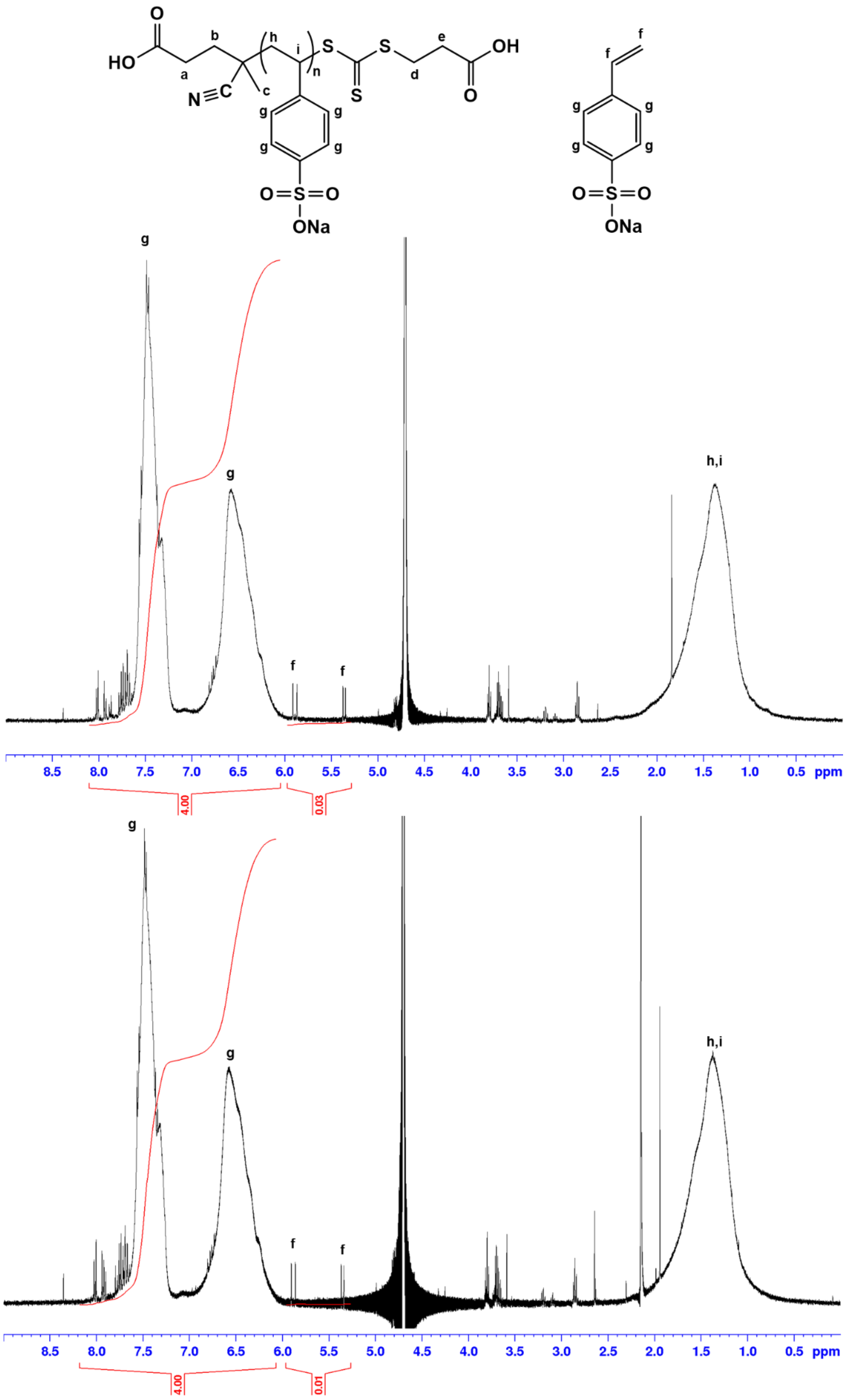

Figure S4: ^{1}H NMR spectra of 40 kDa PSS-CTA (top) and 80 kDa PSS-CTA (bottom) in D_2O. Red markers indicate integrals boundaries and corresponding integral values.

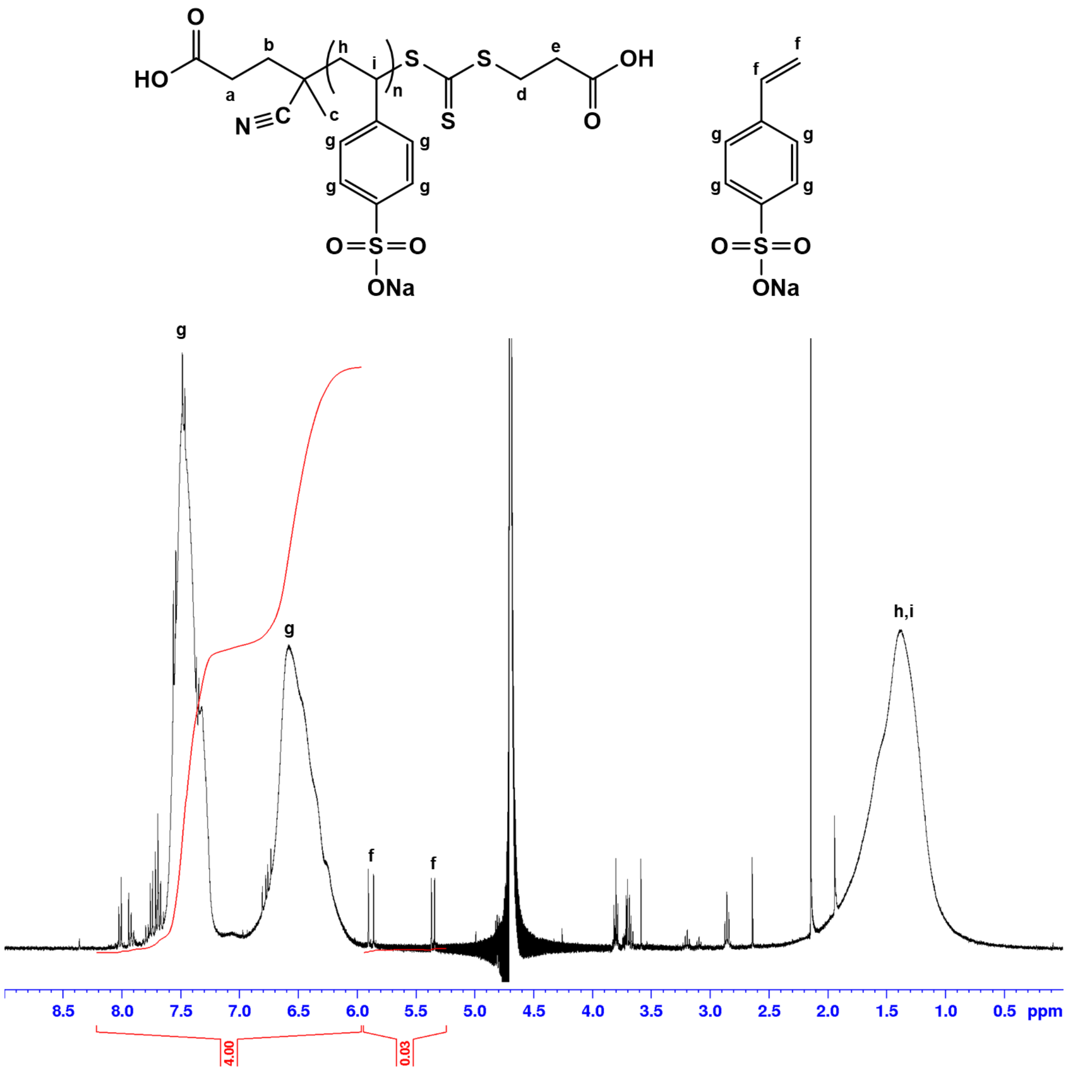

Figure S5: ^{1}H NMR spectra of 160 kDa PSS-CTA in D_2O. Red markers indicate integrals boundaries and corresponding integral values.

Table S2: NMR and SEC characterization of PSS-CTAs synthesized by aqueous RAFT polymerization of SSNa in water.

PSS-CTA	$[SSNa]_0/[CEPCA]_0$	^{1}H NMR			SEC (RI, Water)			
		Monomer Conversion (%)	$M_{n,\ NMR\ Conversion}$[a] (Da)	$M_{n,\ NMR}$[b] (Da)	$M_{n,\ SEC}$ (Da)	$M_{w,\ SEC}$ (Da)	$M_{p,\ SEC}$ (Da)	$Đ$
10 kDa PSS-CTA	48	100	10 300	9900	7800	8600	8400	1.09
20 kDa PSS-CTA	97	100	20 300	27 600	14 800	17 200	16 800	1.16
40 kDa PSS-CTA	194	98.5	39 700	n.d.[d]	25 400	32 700	30 200	1.28
80 kDa PSS-CTA	388	99.5	79 900	n.d.	42 100[c]	61 700	71 000	1.46[c]
160 kDa PSS-CTA	776	98.5	157 900	n.d.	91 800[c]	214 000	207 300	2.33[c]

[a] $M_{n,\ NMR\ Conversion} = [SSNa]_0/[CEPCA]_0 \times \alpha \times M_w^{SSNa} + M_w^{CTCPA}$, where $\alpha = (I^{5.3\text{-}5.9\ ppm}/3)/(I^{5.9\text{-}8.1\ ppm}/4)$ where I corresponds to the integrated value obtained between lower and upper bounds designated in superscript.

[b] $M_{n,\ NMR} = (I^{5.9\text{-}8.1ppm}/4)/(I^{3.2\text{-}3.6ppm}/2)$ where $I^{5.9\text{-}8.1ppm}$ corresponds to the phenyl group protons in SSNa and $I^{3.2\text{-}3.6ppm}$ corresponds to the α-CH_2 to the thiocarbonylthio group. $I^{3.2\text{-}3.6ppm}$ peaks are close to the baseline for PSS-20kDa, and not easily observed for higher molecular weight PSS-CTAs.

[c] Dispersity and $M_{n,\ SEC}$ for high molecular weight PSS are difficult to determine due to strong buffer effects causing low molecular weight tailing (**Figure S7**). The tailing of these polymers was attributed to buffer effects as identical tailing was observed in the SEC traces in high molecular weight PSS calibration standards.

[d] Not determined (n.d.) entries occur as $I^{3.2\text{-}3.6ppm}$ proton peaks are close to the baseline in higher molecular weight PSS-CTA spectra.

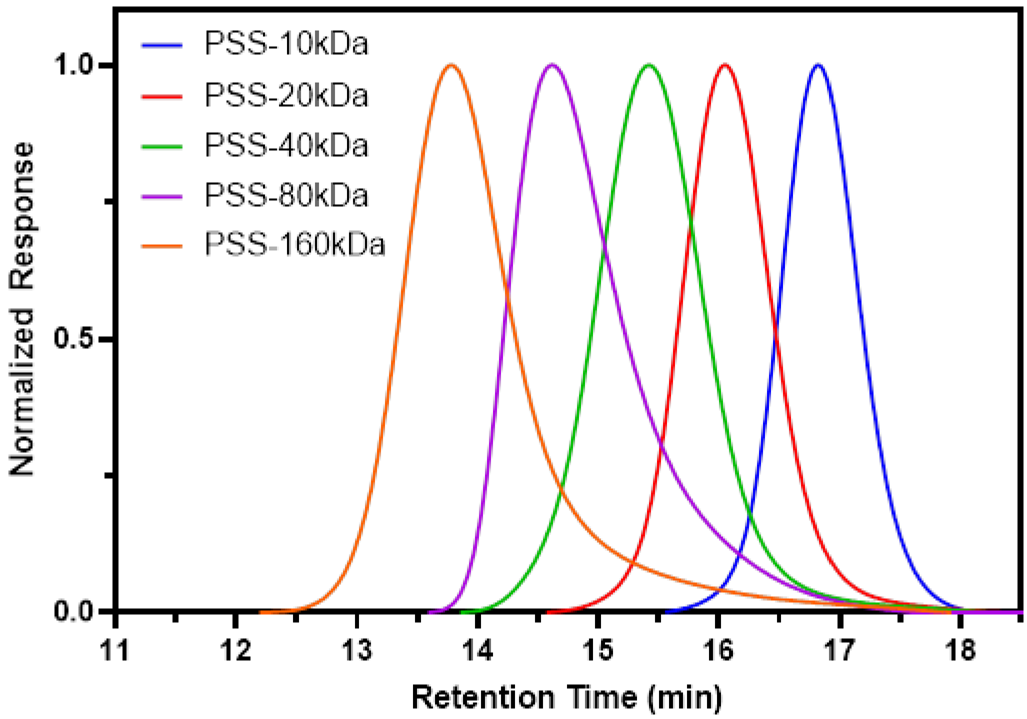

Figure S6. Size exclusion chromatography traces of PSS-CTAs after purification.

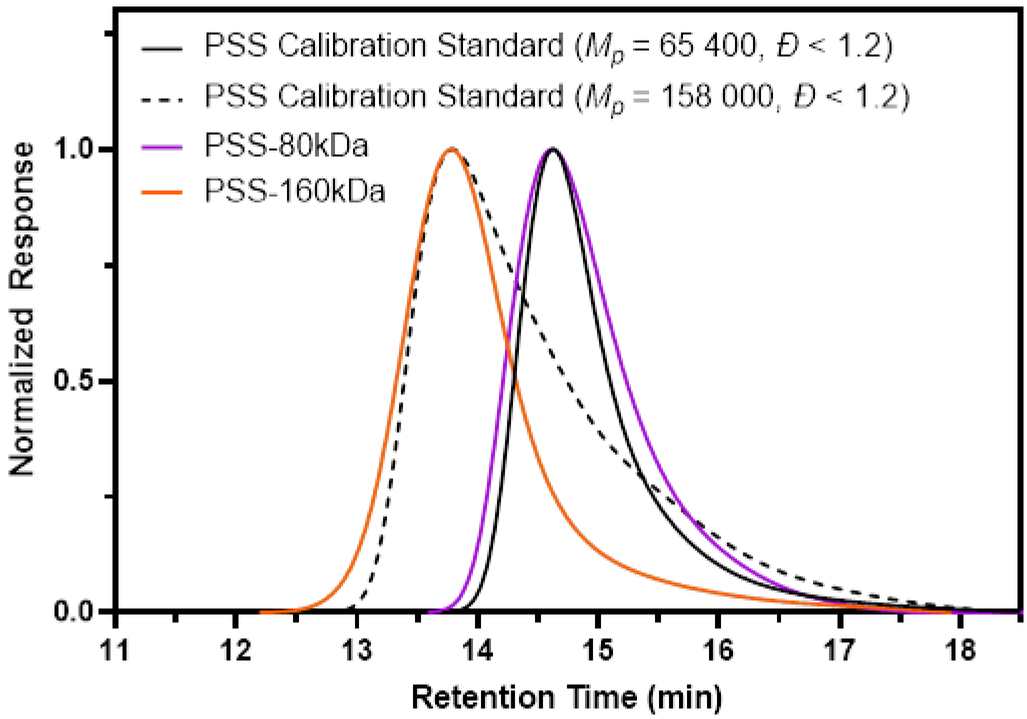

Figure S7. Size exclusion chromatography traces of PSS-80kDa and PSS-160kDa compared to the high molecular weight polystyrene sulfonate calibration standards. For high molecular weight PSS, significant low molecular weight tailing was observed for both synthesized PSS-CTAs and PSS standards, which are attributed to buffer effects. This tailing affects the reported dispersity and *M*, which were significantly different from the calibration specifications ($Đ < 1.2$). The dispersity values of the M_p = 65 400 Da and 158 000 Da calibration standards measured by SEC were $Đ = 1.58$ and Đ = 2.92, respectively.

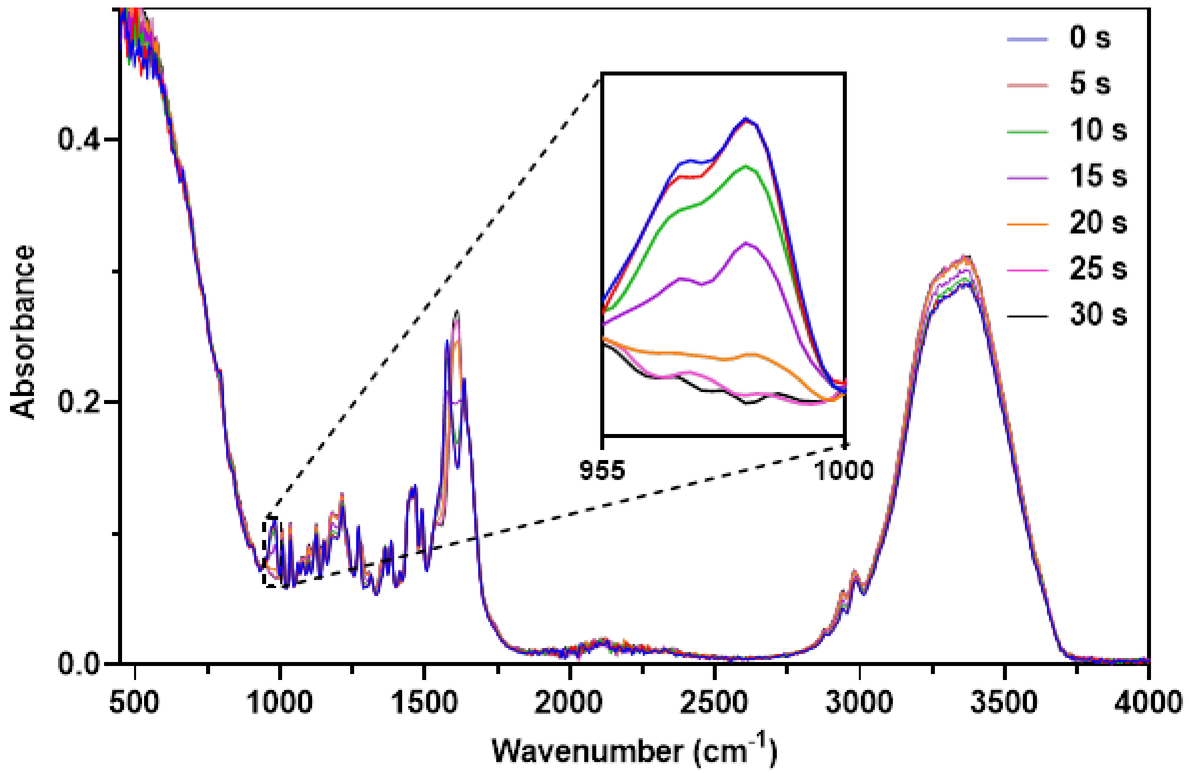

Figure S8. FTIR spectra of IEX-10kDa during irradiation with 405 nm light. Inset shows the peak at ~ 955 – 1000 cm^{-1} where all absorbance in this range is attributed to the C=C bending mode of the vinyl bonds in both MBAm and DEAm.

Table S3. 3D printing parameters for resin formulations.

Formulation	Slicing Layer Thickness (μm)	Layer Cure Time (s)
IEX-10kDa	50	40
IEX-20kDa	50	22
IEX-40kDa	50	18
IEX-80kDa	50	16
IEX-160kDa	50	14
IEX-20kDa + 0.01 wt.% Sudan II	50	25
IEX-80kDa + 0.01 wt.% Sudan II	50	25

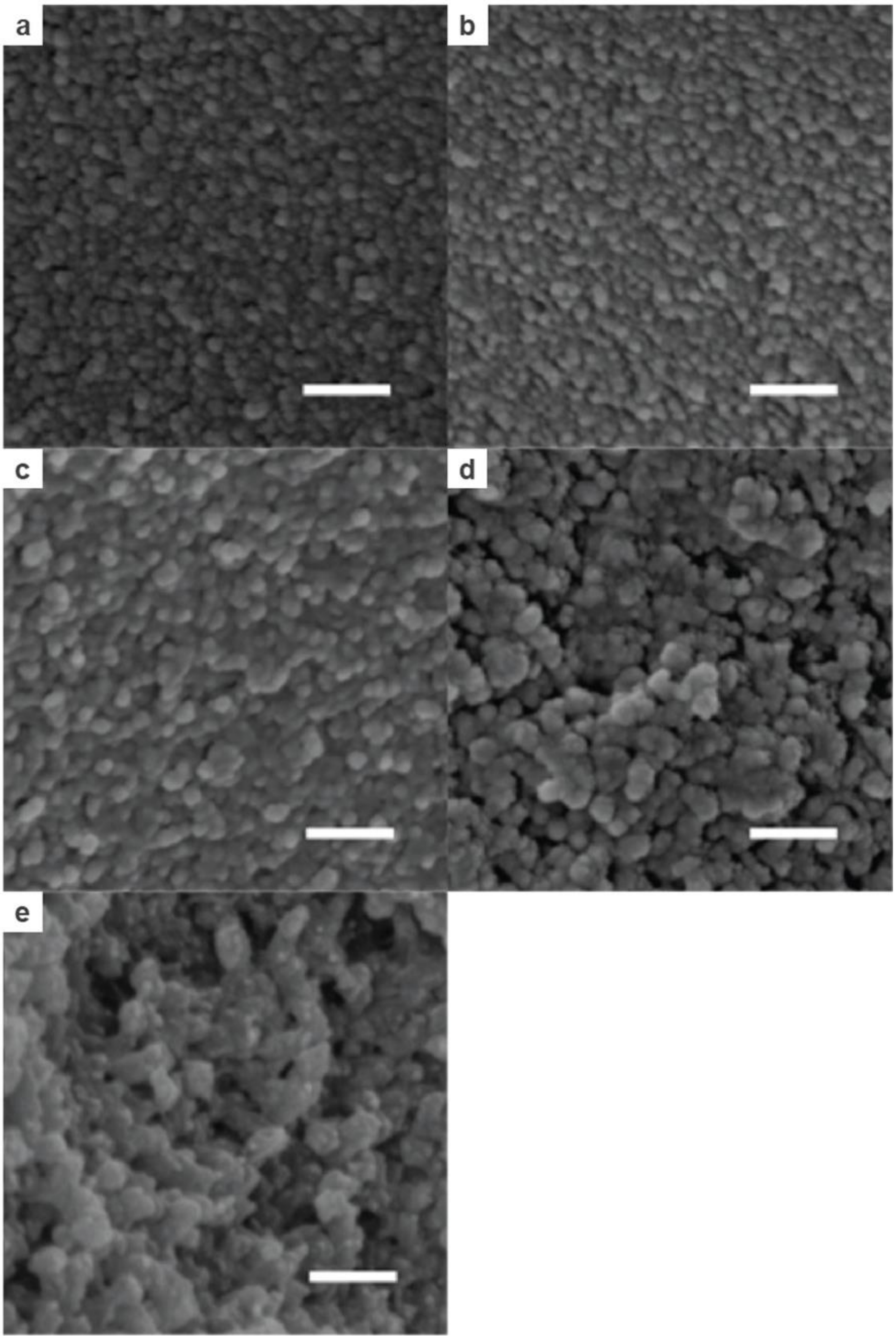

Figure S9. SEM micrographs of PIMS materials after freeze-drying. a) IEX-10kDa; b) IEX-20kDa; c) IEX-40kDa; d) IEX-80kDa; e) IEX-160kDa. Scale bar indicates 100 nm.

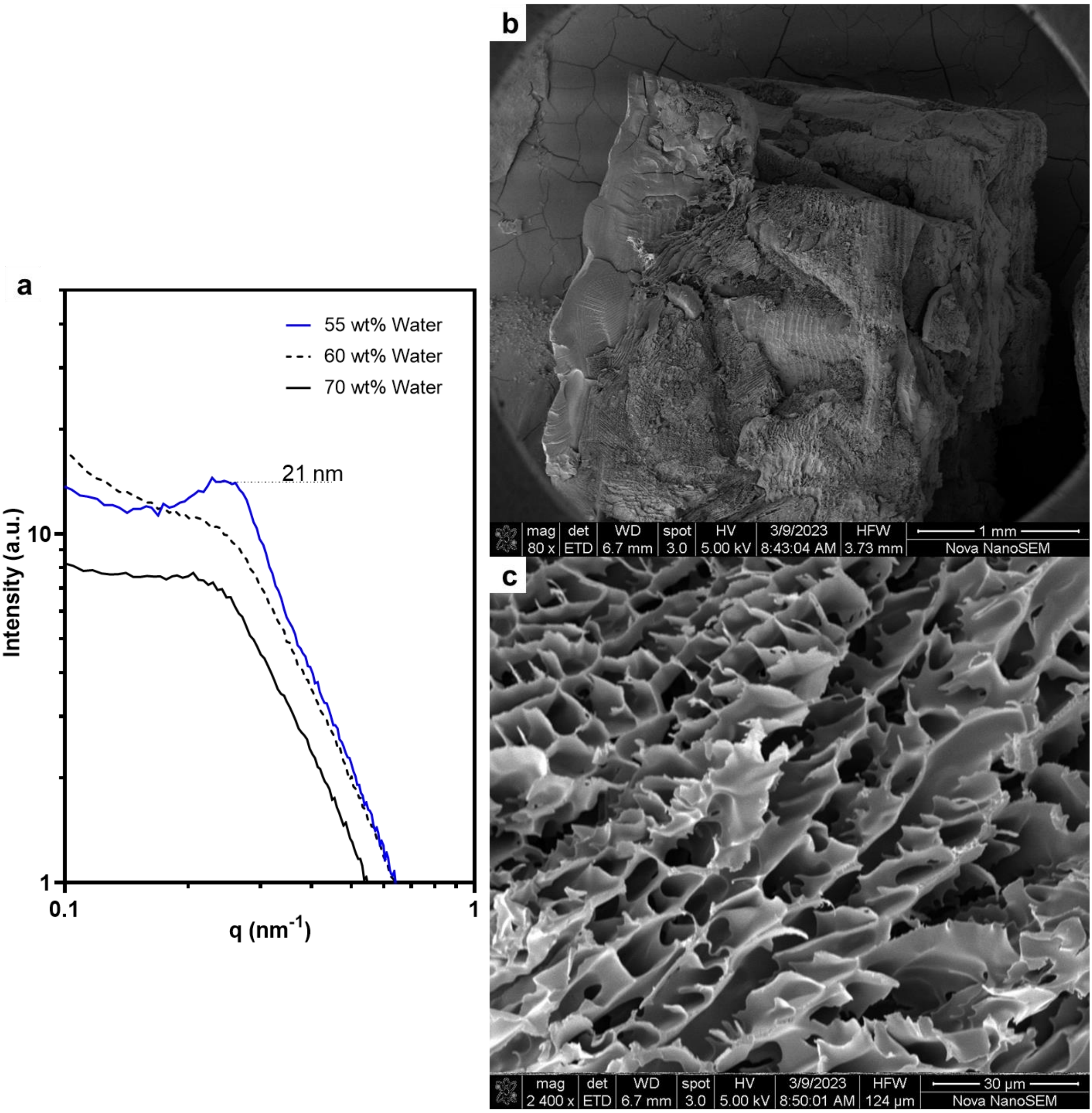

Figure S10. Characterization of IEX materials with high water content. a) SAXS profiles of IEX-20kDa with increasing water content, exhibiting a loss in intensity of the correlation peak at 21 nm and appearing as a broad shoulder rather than a sharp peak; b) and c) SEM of a freeze-dried IEX-20kDa sample with 70 wt.% water showing some structural differences to 55 wt% water in **Figure S9**.

Table S4. BET specific surface area from nitrogen sorption experiments. Despite some similarities in of SEM images to porous materials in literature,[4-8] BET analysis shows negligible pore volume and surface area.

Formulation Name	Surface Area (m^2/g)
IEX-20kDa	14.5
IEX-80kDa	11.6
IEX-160kDa	16.9
IEX-20kDa, 70 wt.% Water	17.7

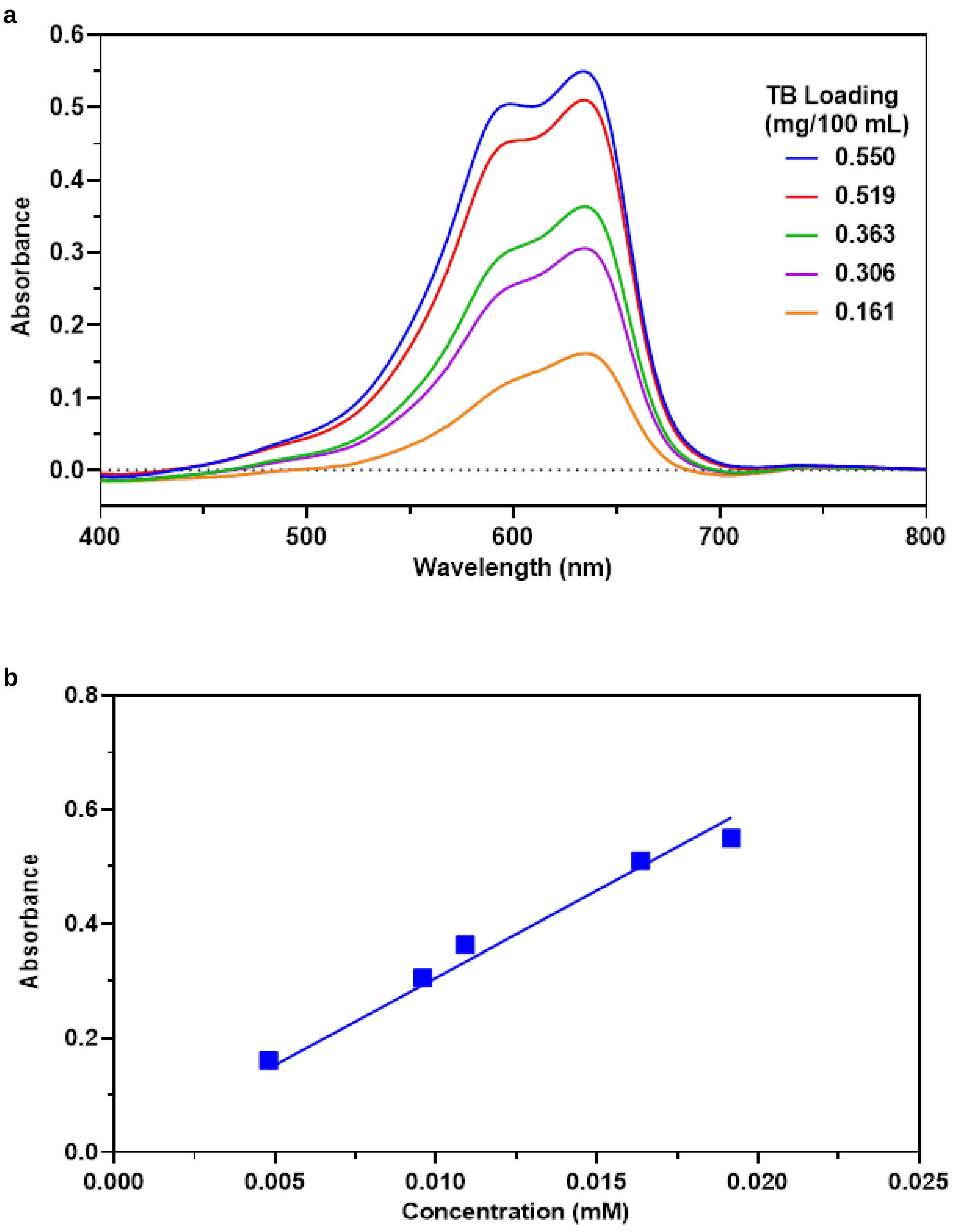

Figure S11. Absorbance of toluidine blue (TB) in pH = 7.4 phosphate buffer using a 1 cm quartz cuvette. a) UV-VIS spectra of TB solution at different concentrations; b) calibration curve derived from a), line through the points is a fit to Beer-Lambert law (R^2 = 0.997), where the molar extinction coefficient is ε = 30518 M^{-1} cm^{-1}.

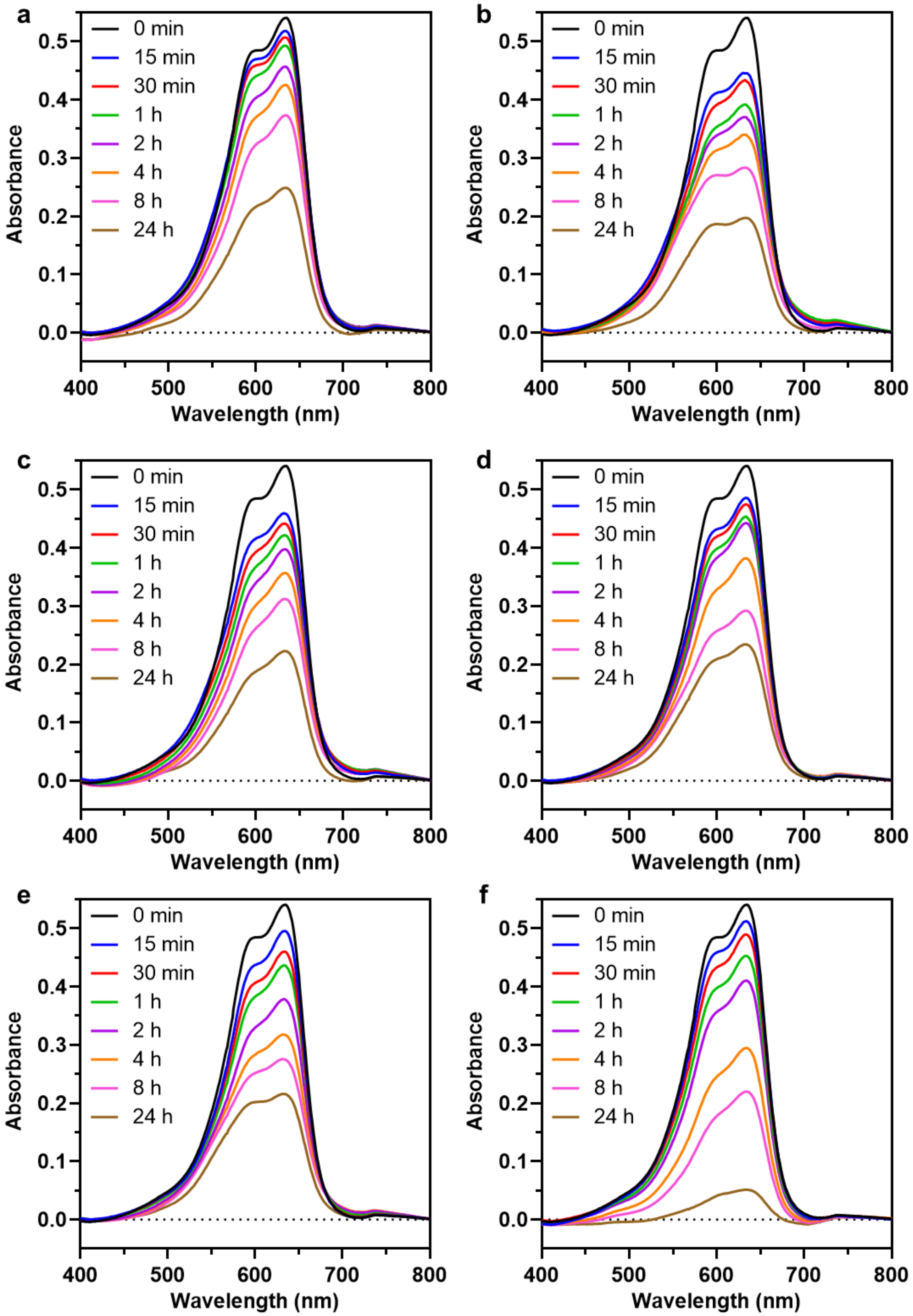

Figure S12. Typical time resolved UV-VIS absorption spectra for a 0.5 mg/100 mL solution of TB in pH = 7.4 phosphate buffer for samples containing a) IEX-10kDa; b) IEX-20kDa; a) IEX-40kDa; a) IEX-80kDa; a) IEX-160kDa; a) Amberlite IRC120Na.

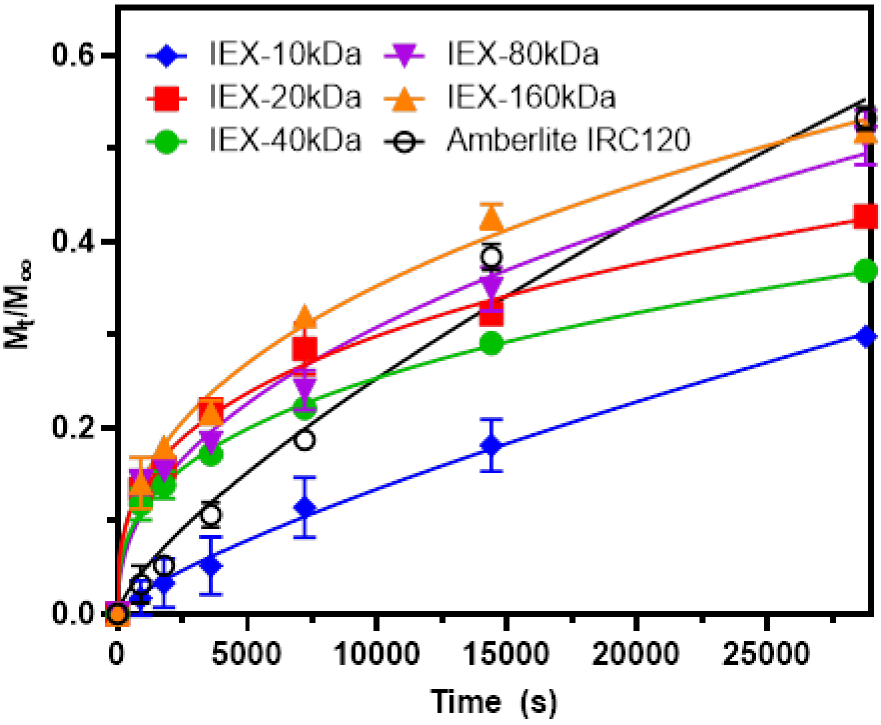

Figure S13. Time resolved dye uptake . Lines through points are a fit of the Korsmeyer-Peppas model. Model parameters are shown in Table S5.

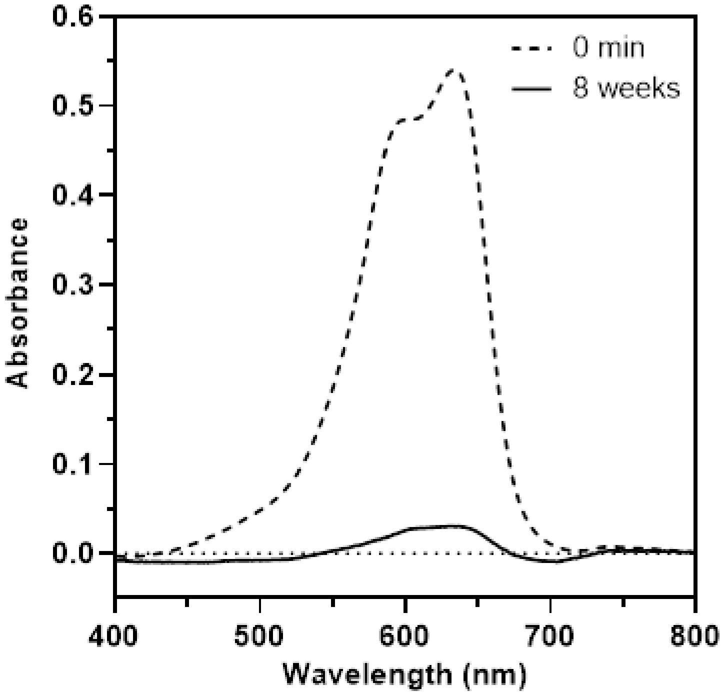

Figure S14. UV-VIS absorption spectra for IEX-20kDa in 0.5 mg/100 mL of TB in pH = 7.4 phosphate buffer shown initially and at 8 weeks, where absorbance corresponded to 1183 μg/g TB removal.

Table S5. Korsmeyer Peppas model parameters.

Formulation name	*k* (s^{-1})	*n*	R^2
IEX-10kDa	0.00011	0.76	0.96
IEX-20kDa	0.014	0.33	0.99
IEX-40kDa	0.010	0.35	0.99
IEX-80kDa	0.0050	0.44	0.97
IEX-160kDa	0.0010	0.38	0.99
Amberlite IRC 120 Na	0.00028	0.74	0.98

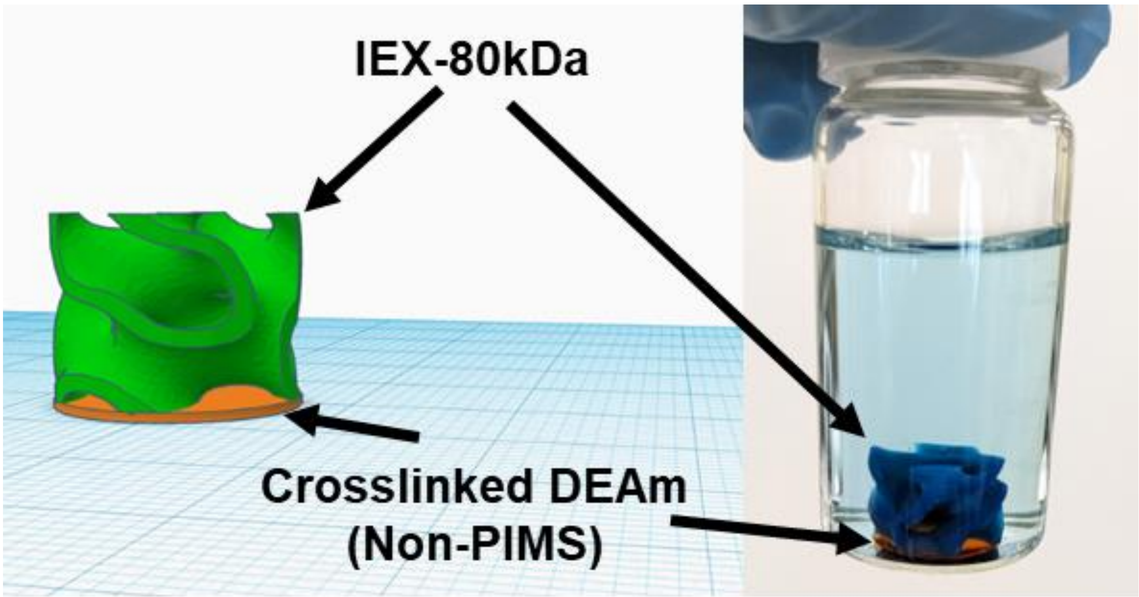

Figure S15. Illustration of the 3D model used for the cylindrical gyroid. The base portion of the gyroid is printed from a non-PIMS crosslinked DEAm to provide better adhesion to the build platform during printing of larger objects. During the dye uptake experiment, this region also serves as a non-PIMS control. After submerging in TB solution for 24 h, the non-PIMS crosslinked DEAm section was translucent and red due to the presence of Sudan II, and no blue coloration was observed. This is expected as crosslinked DEAm is incompatible with water, and neither water nor TB is expected to diffuse into the DEAm bulk. The portion of the gyroid printed with IEX-80kDa strongly retains a blue coloration from the absorbed TB from solution.

References

[1] Leach, J.B. and C.E. Schmidt, *Characterization of protein release from photocrosslinkable hyaluronic acid-polyethylene glycol hydrogel tissue engineering scaffolds.* Biomaterials, 2005. **26**(2): p. 125-135.

[2] ChemicalBook. *Poly(sodium 4-styrenesulfonate)*. 2024 [Accessed 2024 Jan]; Available from: https://www.chemicalbook.com/ChemicalProductProperty_EN_CB3158410.htm.

[3] Bae, Y.H., T. Okano, and S.W. Kim, *Temperature dependence of swelling of crosslinked poly(N,N′ -alkyl substituted acrylamides) in water.* J. Polym. Sci., Part B: Polym. Phys., 1990. **28**(6): p. 923-936.

[4] Saba, S.A., M.P.S. Mousavi, P. Bühlmann, and M.A. Hillmyer, *Hierarchically Porous Polymer Monoliths by Combining Controlled Macro- and Microphase Separation.* J. Am. Chem. Soc., 2015. **137**(28): p. 8896-8899.

[5] Seo, M., S. Kim, J. Oh, S.-J. Kim, and M.A. Hillmyer, *Hierarchically Porous Polymers from Hyper-cross-linked Block Polymer Precursors.* J. Am. Chem. Soc., 2015. **137**(2): p. 600-603.

[6] Xie, Y. and M.A. Hillmyer, *Nanostructured Polymer Monoliths for Biomedical Delivery Applications.* ACS Appl Bio Mater, 2020. **3**(5): p. 3236-3247.

[7] Seo, M. and M.A. Hillmyer, *Reticulated Nanoporous Polymers by Controlled Polymerization-Induced Microphase Separation.* Science, 2012. **336**(6087): p. 1422-1425.

[8] Peterson, C.H., J.R. Werber, H.K. Lee, and M.A. Hillmyer, *Tailored Mesoporous Microspheres by Polymerization-Induced Microphase Separation in Suspension.* ACS Appl. Polym. Mater., 2022. **4**(6): p. 4219-4233.
